# Pan-cancer analysis of genomic properties and clinical outcome associated with tumor tertiary lymphoid structure

**DOI:** 10.1038/s41598-020-78560-3

**Published:** 2020-12-09

**Authors:** Ziying Lin, Lixia Huang, ShaoLi Li, Jincui Gu, Xiaoxian Cui, Yanbin Zhou

**Affiliations:** 1 Department of Respiratory and Critical Care Medicine, The First Affiliated Hospital of Sun Yat-sen University, Guangzhou, China; 2 Department of Respiratory Medicine, The 8th Affiliated Hospital of Sun Yat-sen University, Shenzhen, China

**Keywords:** Cancer microenvironment, Tumour immunology

## Abstract

How the genomic landscape of a tumor shapes the formation of tertiary lymphoid structure (TLS) and how might TLS alter the clinical outcome or response to immunotherapy had not been systematically explored. Utilizing the genomic and transcriptome data of solid tumors on TCGA, we quantified TLS based on a previous identified 12-chemokine signature and evaluated its correlation with mutation/neoantigen burden, functional mutation of oncogenes and the presence of viral infection. Clinical data was integrated to decide the prognostic significance of TLS for different cancers after surgical treatment. Publicly available data (clinical and transcriptome data) of immunotherapy clinical trials involving melanoma and lung cancer were also collected to evaluate TLS’s association with therapeutic outcome. Mutation burden and predicted neoantigen counts were positively correlated with TLS scoring in multiple cancer types. Mutation in tumor suppressor genes (KEAP1, PBRM1) and genes involved in extrinsic apoptosis (CASP8), antigen-presentation (HLA-A, HLA-B), immune regulation (SMAD4) or DNA repair (BRCA1, BRCA2, TP53BP1) correlated with TLS alteration in multiple tumor types, indicating the interaction between mutation landscape and TLS formation. Epstein-Barr virus (EBV) infection in gastric cancer and human papillomavirus (HPV) infection in Head and Neck squamous cell carcinoma were associated with increased TLS scoring. High TLS scoring predicted favorable prognosis in certain cancer after surgical treatment and improved response to immunotherapy in lung cancer and melanoma. Our findings unraveled the genomic properties associated with TLS formation in different solid tumors and highlighted the prognostic and predictive significance of TLS in surgical treatment and immunotherapy.

## Introduction

Tertiary lymphoid structures (TLS), also termed ectopic lymphoid organs, are the lymphoid tissue harboring architecture highly analogies that of the secondary lymphoid organs (SLOs)^1^. While SLOs are the constitutive lymphoid tissue that develop during embryogenesis, TLS only develop in non-lymphoid tissue with persistent inflammation, like sites with chronic infection, organs transplantation, autoimmune diseases and cancers^2,3^. TLS exhibit all the characteristic structures associated with the generation of adaptive immune response, which include a germinal center with proliferating B cells and follicular dendritic cells, a peripheral T cell zone interacting with mature dendritic cells, and high endothelial venules (HEV) that allows the extravasation of naïve T and B cells^4,5^. Being the sites for the generation of circulating effector immune cells, TLS constitute a paramount component of the tumor microenvironment (TME) and play a key role in controlling tumor growth.

The presence of TLS had been reported to be associated with favorable prognosis in multiple solid tumors^6–9^, which might be associated with their capacity in inducing a long-lasting antitumor response. Also, TLS of different spatial distribution might convey different prognostic significance, as previous studies reported that TLS in adjacent normal liver tissue correlated with increased risk of tumor relapse or had no prognostic value, while TLS in the tumor core of hepatocellular carcinoma predicted a favorable outcome^10,11^. In the era of cancer immunotherapy, where unprecedent tumor growth control can be achieved immune checkpoint blockade (ICB)^12^, TLS have attracted more and more attention as an unique structure of the tumor immune microenvironment. The major obstacle of immunotherapy is that only a selected subset of patients response to ICB, and the determination of accurate biomarkers for response is urgently in need^13^. Since the identification of immune cell infiltration, neoantigen burden to be predictive of response to ICB, emerging evidences also show a significant correlation between TLS density and therapeutic efficacy of immunotherapy^14,15^. All the exist evidences support the proposition that TLS may serve as a critical marker in classifying tumors microenvironment.

As a complex network comprising different types of immune populations, tumor immune microenvironment (TME) plays an important role in tumor development and progression, and also exerts great impact on prognosis or therapeutic efficacy. While the majority of previous studies on TME highlighted the significance of individual immune cell types, TLS as a functional entity that integrating innate and adaptive immune populations involved in anti-tumor response has not been fully investigated in the existent studies. The genomic features associated with TLS formation and its interaction with TME is yet to be elucidated. Its prognostic significance especially with regard to different distributed locations, and its predictive value in therapeutic outcome of immunotherapy in different malignant diseases remain to be verified.

The major challenge in the research of TLS is its detection and quantification. Canonical detecting methods included configuration identification by hematoxylin and eosin (H&E) staining and multiplex labelling of selected markers by immunohistochemistry (IHC)^16,17^, which is inconvenient to quantify and also easily subjected to objective bias. Several gene signatures for the detection of TLS identified from transcriptomic analysis in different human cancers had be proved to be feasible in the quantification of TLS. A 12-chemokine signature is the most widely used signature that had been applied for the quantification of TLS in multiple solid tumors including colorectal cancer, melanoma, hepatocellular carcinoma and breast cancer etc.^18–21^. With the high-dimensional data sets in The Cancer Genome Atlas (TCGA) that involving all types of solid tumors and the previously identified TLS signature, it’s feasible for us to dissect the intrinsic factors and extrinsic factor associated with TLS formation in tumor microenvironment and how it impacts the prognosis in the pan-cancer setting. Correlation between TLS and immunotherapy response could also be verified with publicly available datasets from clinical studies on immunotherapy.

## Results

### Transcriptome analysis of TLS

**Figure 1:**
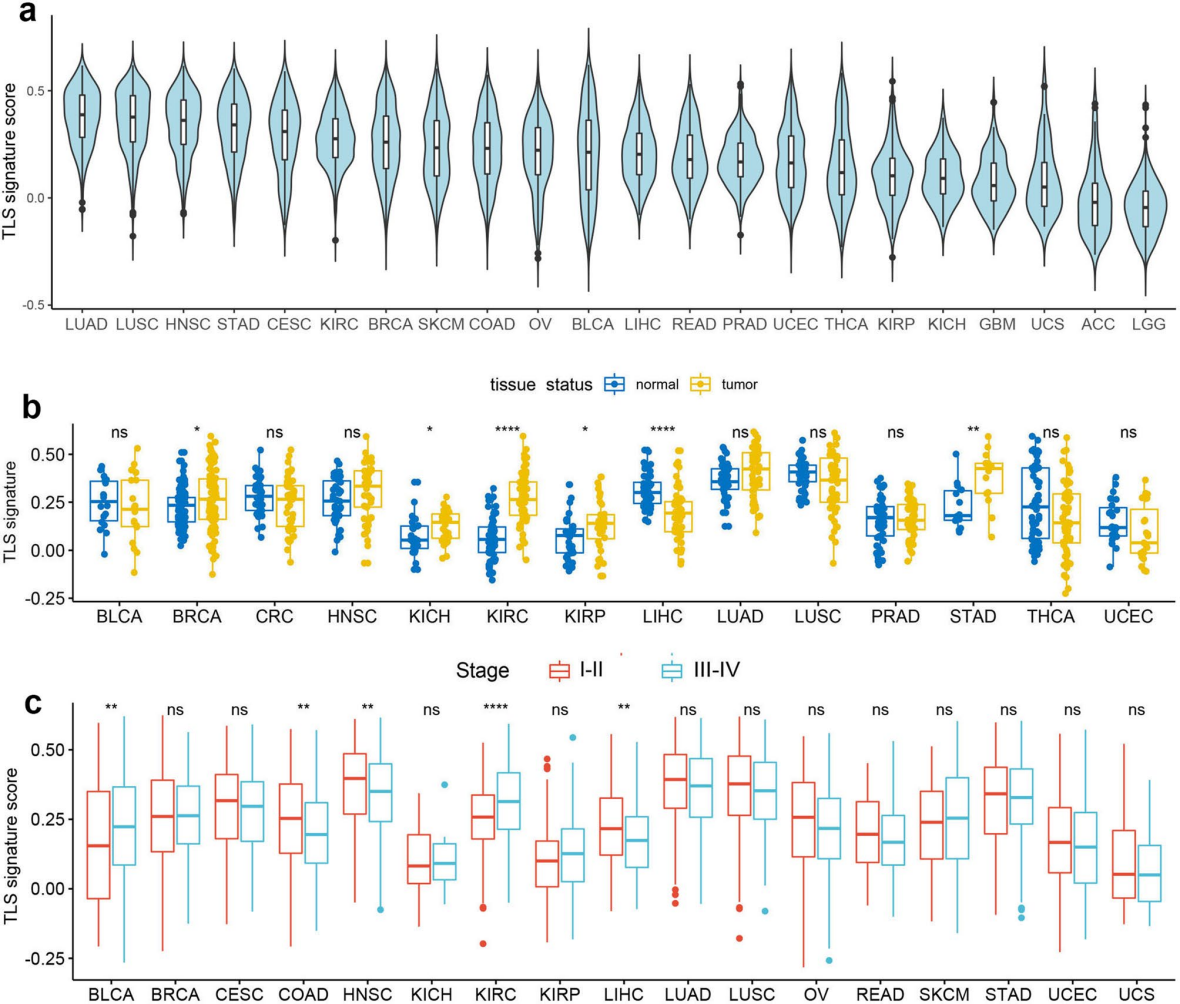
Pan-cancer analysis of TLS based on 12-chemokine signature. (**a**) Expression of TLS signature across 22 solid tumor types; (**b**) Comparison of TLS signature expression between tumor tissue and matched adjacent normal tissue; (**c**) Comparison of TLS signature expression between early-stage tumors (stage I–II) and advanced tumors (stage III–IV). *P < 0.05; **P < 0.01; ***P < 0.001; ****P < 0.0001; ns, not significant.

RNA-seq raw data of 8672 tumor samples and 619 adjacent normal tissue samples were pooled and preprocessed to obtain expression data comparable across samples of different tumor types. TLS enrichment score of each samples was calculated based on a 12-chemokine gene signature derived from metagene correlated with enrichment of TLS in colorectal cancer and also proved to be robust and specific in predicting TLS abundance in other tumor types like melanoma, breast cancer and lung cancer^18–21^. Cancer type-specific expression of the TLS signature revealed a dramatic heterogeneity of TLS abundance across different solid tumors (Fig. 1a). High expression of TLS signature was observed in lung adenocarcinoma (LUAD), lung squamous cell carcinoma(LUSC), head and neck squamous cell carcinoma (HNSC), and stomach adenocarcinoma (STAD), indicating the existence of abundant TLS. Adrenocortical carcinoma (ACC) and lower grade glioma (LGG) demonstrated extreme low expression of TLS associated chemokines, hinting the absence of TLS within tumor mass.

We also evaluated the spatial distribution of TLS by comparing the expression of 12-chemokine signature between tumors and the paired normal tissue samples. As shown in Fig. 1b, significant higher expression of TLS signature was observed in adjacent normal tissue as compared to tumor tissue for multiple cancer types including breast cancer (BRCA), kidney chromophobe (KICH), kidney renal clear cell carcinoma (KIRC), kidney renal papillary cell carcinoma (KIRP) and STAD. Of note, the opposite finding was observed in liver hepatocellular carcinoma (LIHC), where tumor tissue expressed significant higher TLS associated chemokines compared to the adjacent normal tissue. No significant difference between tumor and adjacent normal tissue was observed for the rest solid tumors except for ACC, cervical squamous cell carcinoma and endocervical adenocarcinoma (CESC), SKCM, glioblastoma (GBM) and LGG, which were not applied to this analysis due to the lack of matched normal tissue.

We didn’t observe a clear-cut correlating pattern between TLS signature expression and tumor staging (Fig. 1c). While advanced tumor of bladder carcinoma (BLCA) and kidney renal clear cell carcinoma (KIRC) had significant higher TLS scoring as compared to early stage tumor, the opposite finding was observed in colon adenocarcinoma (COAD), HNSC and LIHC, and no correlation between staging and TLS enrichment was observed for the other solid tumors.

**Figure 2:**
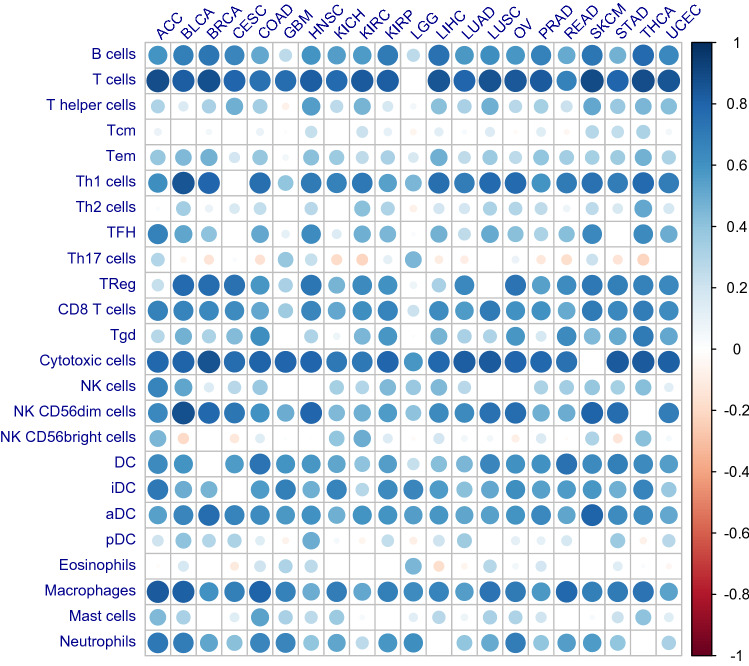
Correlation between expression of TLS signature and infiltration of immune cells. The Correlation between the enrichment score of TLS signature and 24 types of infiltrating immune cells was evaluated by Pearson Correlation analysis. Node color is determined by correlation, and node size indicates the significance of correlation. Only nodes with Correlation significance (false discovery rate < 0.05) were shown.

We further evaluated the correlation between TLS signature expression and the cellular composition of TME. As shown in Fig. 2, TLS scoring demonstrated strong correlation (R > 0.6) with the infiltrating level of B cells, T cells, T helper type 1 (Th1) cells, cytotoxic T cells, DC, macrophages etc. across most of the tumor types. All these immune cells that demonstrated the most significant correlation with TLS signature expression were considered as the major components of TLS, suggesting that expression score of the 12-chemokine signature can efficiently reflect the enrichment of TLS in TME.

### TLS and oncogenic viral infection

**Figure 3:**
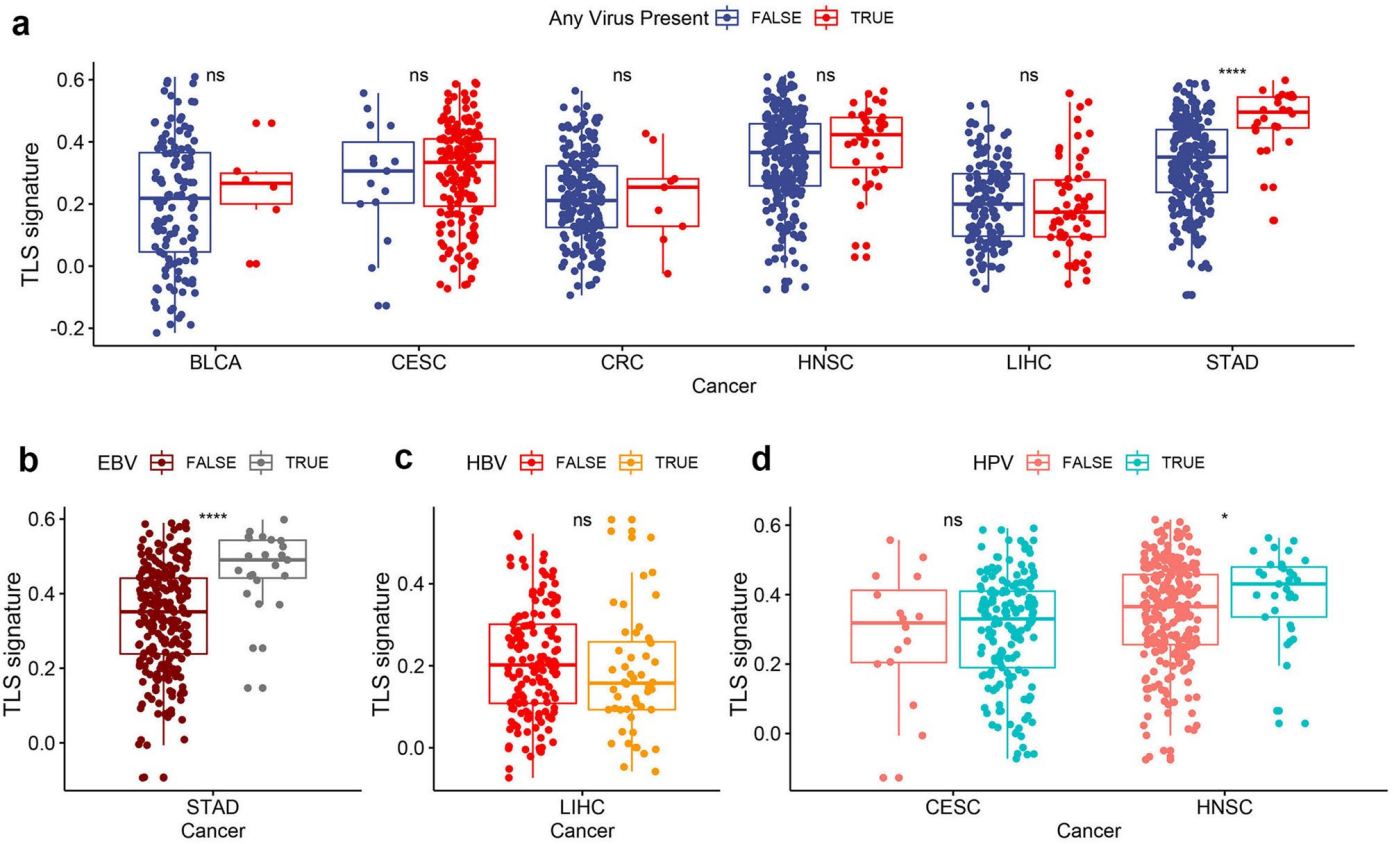
Viral Infection Is Tumor-Specific and Associated with Higher TLS scoring in a Subset of Tumor Types. (**a**) Association between TLS scoring and the presence of any viral infection in different tumor types; (**b)** association between TLS scoring and Epstein-Barr virus (EBV) infection in gastric cancer (STAD); (**c**) association between TLS scoring and Epstein- Hepatitis B (HBV) infection in liver cancer (LIHC); (**d**) association between TLS scoring and Epstein- Hepatitis B (HBV) infection in human papillomavirus (HPV) infection in cervical cancer (CESC) Head and Neck squamous cell carcinoma (HNSC). *P < 0.05; **P < 0.01; ***P < 0.001; ****P < 0.0001; ns, not significant.

Viral infection accounts for the oncogenesis of a subset of malignancies and also associates with the formation of TLS. Whether the presence of virus within the tumor contribute to the development of TLS remains unknown. Association between viral infection and TLS density was decided by comparing the TLS scoring between samples with and without viral infection in each tumor types, which was detected by the presence of viral genome within the tumor mass. Among all the tumor sample applied to the viral detection, the presence of viral genome was frequently observed in certain tumor types including BLCA, CESC, CRC, HNSC, LIHC and STAD. Virus detected in CESC and HNSC was predominated by human papillomavirus (HPV), whereas Hepatitis B (HBV) and Epstein-Barr virus (EBV) was highly prevalent in LIHC and STAD respectively. As shown in Fig. 3a, the presence of viral infection was only significantly associated with increased TLS scoring in STAD. As for specific virus types, EVB and HPV infection significantly correlated with high expression of TLS signature in STAD and HNSC respectively (Fig. 3b, d). It’s worth mention that HPV infection exhibited no significant association with TLS scoring in CESC, neither did EBV in LIHC (Fig. 3c,d).

### TLS enrichment is positively correlated with mutation/neoantigens burden

**Figure 4:**
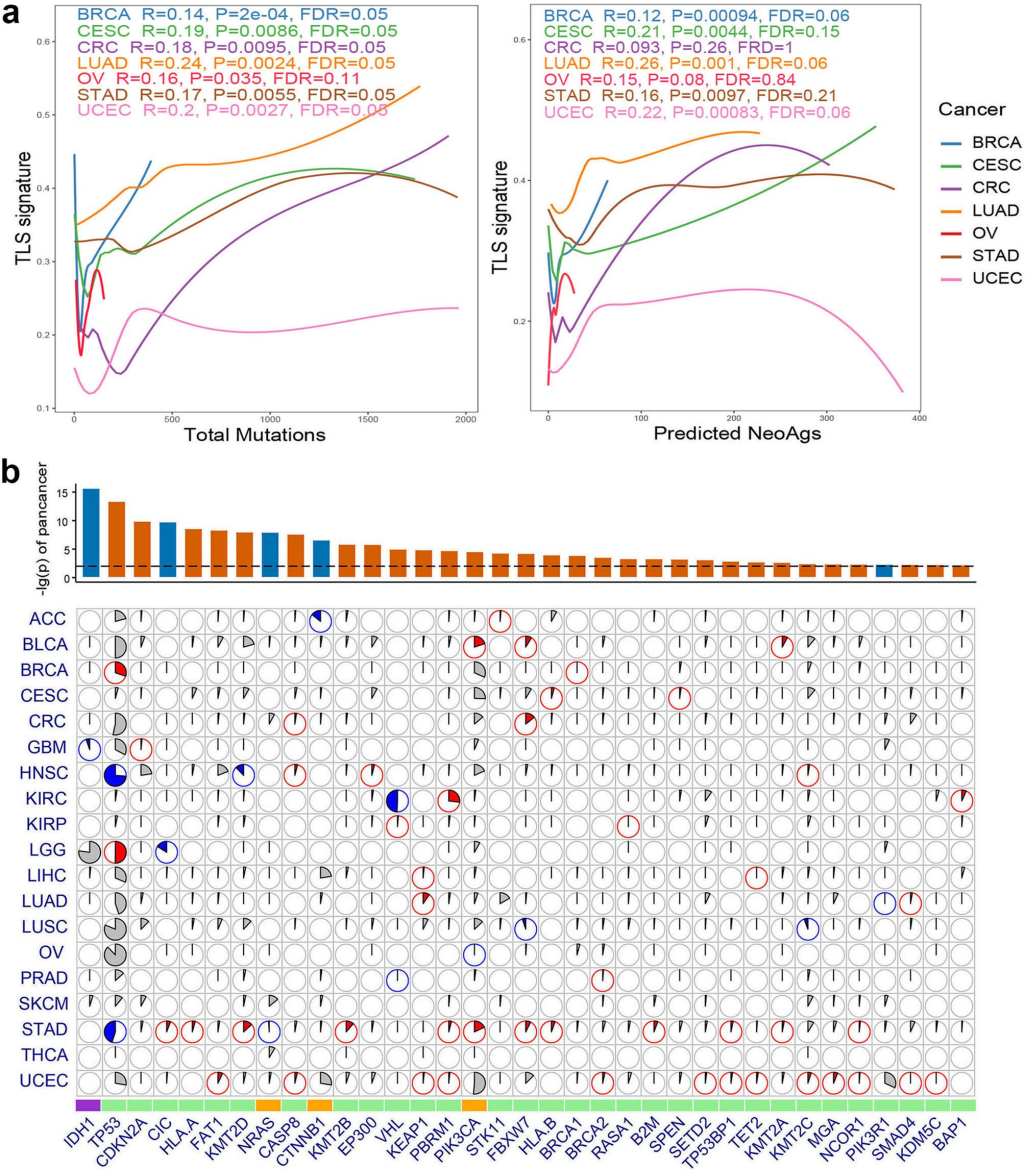
Correlation of TLS scoring with mutation/neoantigen loads and driver gene mutations. (**a**) Local regression curves showing significant relationships of TLS scoring with total mutation count (left) and predicted neoantigen load (right) in seven tumor types; R value, *p* value and false discovery rate (FDR) of Spearman rank correlation analysis of each tumor type (denoted as lines in different color) were presented; curves span the 5th to 95th percentile of the mutation/neoantigen counts variable; colors correspond to tumor type and are the same in two graphs. (**b**) Driver gene mutations that associated with increased or decreased expression of TLS signature. Only genes showing significance (adj. *P* < 0.01, red for positive, blue for negative association) for non-silent mutation association with TLS scoring in pan-cancer dataset are shown in the top bar plot, with the value of y axis representing log10(*p* value), and the dashed line indicating threshold *P* = 0.01. The pie plots below demonstrated association between TLS scoring and mutation of each driver gene in different tumor types (*P* < 0.05, red for positive, blue for negative and gray for non-significant association). The wedges with color represent the share of samples exhibiting driver mutation.

As the primary site of tumor antigen presentation and the subsequent stimulation of effector immune cells, whether the formation of TLS is associated with the production of tumor neoantigens is yet to be elucidated. Utilizing the mutation information derived from DNA sequencing data in TCGA, we tested the correlation of TLS scoring with mutation burdens as well as the counts of predicted neoantigens. The predicted neoantigen number positively correlated with mutation counts, and their abundance in different tumor types differ from one another (sFig 1). Intriguingly, tumors with high mutation counts or neoantigen load like SKCM, LUSC and LUAD, also demonstrated highly expression TLS signature as previously mentioned (Fig. 1a). Despite the inter-tumor heterogeneity in mutation burden or neoantigen load, both metrics (mutation/neoantigen burden) exhibited significant positive correlation with TLS scoring in multiple tumors including BLCA, BRCA, CESC, LUAD, STAD and uterine corpus endometrial carcinoma (UCEC) (*P* < 0.05) (Fig. 4a). TLS scoring in ovarian cancer (OV) and colorectal cancer (CRC) exhibited significant positive correlation with mutations counts (*P* < 0.05), although its correlation with predicted neoantigen load lacked of statistical significance (*P* > 0.05) (Fig. 4a). Of note, a negative correlation between TLS scoring and mutation/neoantigen burden was also observed in individual tumor types including KIRC (*P* < 0.05 for mutation and neoantigen counts) and thyroid carcinoma (THCA) (*P* < 0.05 for neoantigen counts) (sFig 2).

### TLS and driver gene mutations

Genomic alteration of certain oncogenic pathway of tumor cells can help shape the immune microenvironment as the intrinsic factors. Also, immune microenvironment can exert survival stress on the tumor cells to render the selection of somatic mutation of immune escaping function. We therefore asked whether driver gene mutations were associated with TLS density in tumor microenvironment. By focusing on the known driver genes with annotated mutation information (gain-of-function, loss-of-function, switch-of-function), we first identified 35 driver genes whose mutation significantly correlated with alteration in TLS signature expression in pan-cancer (*P* < 0.01). We further evaluated the association between the mutation status of these genes and TLS scoring in different tumor types (Fig. 4b). Loss-of-function mutation in genes associated with DNA repair like BRCA1, BRCA2, TP53BP1 were correlated with significant higher expression of TLS signature in multiple tumor types, indicating genomic instability might promote the formation of TLS in TME. Higher TLS scoring was also associated with loss-of-function mutations in tumor suppressor genes (KEAP1, PBRM1), genes involved in extrinsic apoptosis (CASP8), antigen-presentation (HLA-A, HLA-B) or immune regulation (SMAD4), indicating the mechanism by which the tumor cells survive or escape the immune surveillance. Mutation in a few genes like IDH1, NRAS, CTNNB1 etc. also demonstrated negative correlation with enrichment score of TLS signature in certain tumor types. Of note, several genes like TP53, PIK3CA, VHL etc. demonstrated discordant correlations with TLS scoring in different tumor types, which might be associated with the different functional status of the genes in different cancers.

### Prognostic impact of TLS in pan-cancer

**Figure 5:**
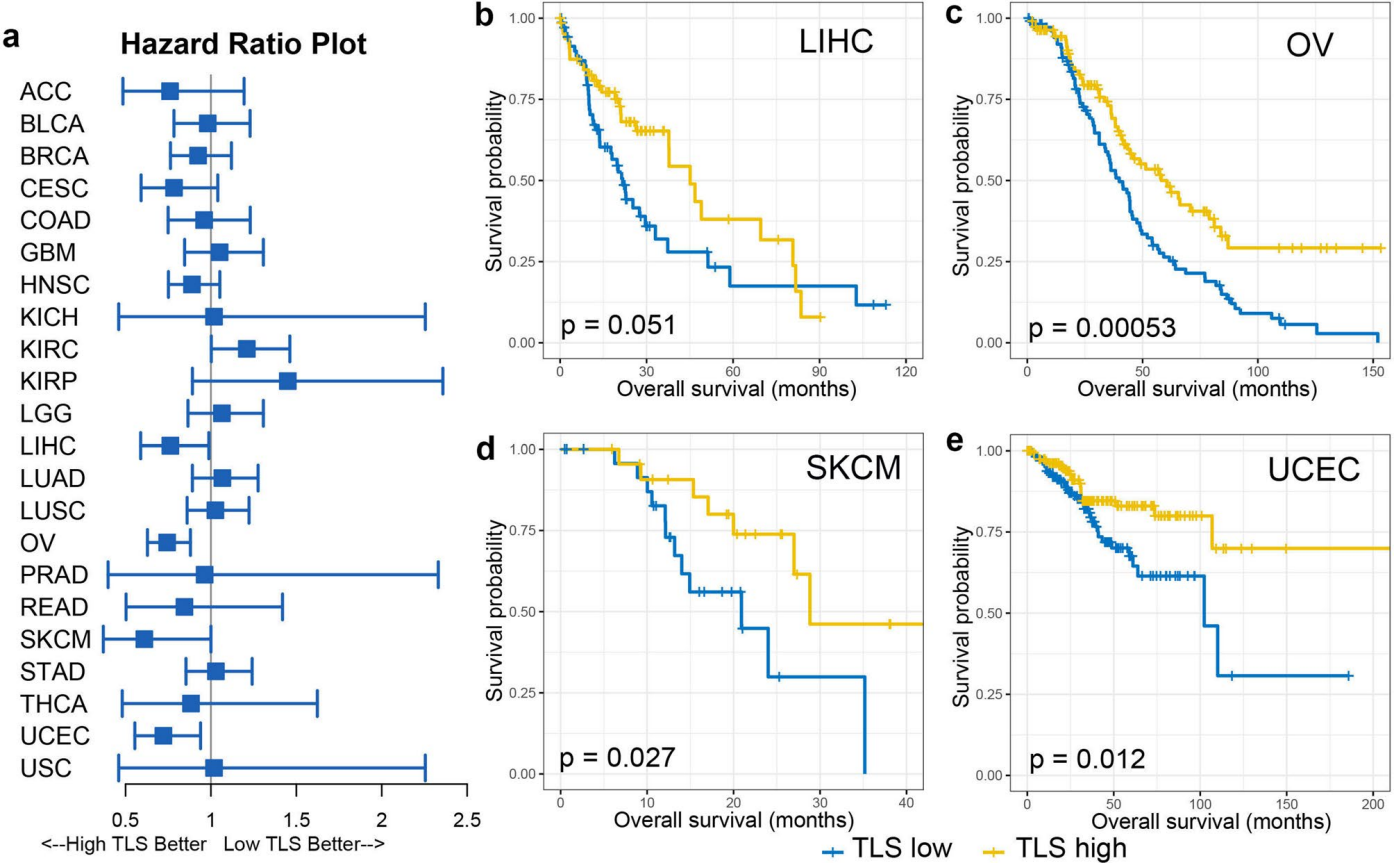
High TLS score associated with improved overall survival in certain tumor types. (**a**) Forest plot demonstrated the survival impact of TLS level for each tumor type. Tumors were categorized into high TLS scoring (> the second tertile), intermediate TLS scoring (between the first and the second tertile) and low TLS scoring (< the first tertile) for each tumor type. Hazard Ratio (5–95% confidence interval) were obtain by cox regression survival analysis. (**b**) Kaplan–Meier plots of overall survival difference between tumors with high TLS scoring and low TLS scoring in LIHC. (**c**) Kaplan–Meier plots of overall survival difference between tumors with high TLS scoring and low TLS scoring in OV. (**d**) Kaplan–Meier plots of overall survival difference between tumors with high TLS scoring and low TLS scoring in SKCM. (**e**) Kaplan–Meier plots of overall survival difference between tumors with high TLS scoring and low TLS scoring in UCEC.

Utilizing the survival data on TCGA, we evaluated the predictive value of TLS signature in the prognosis in cancer patients underwent surgical treatment. Cox regression survival analysis was performed for TLS scoring as well as the infiltrating level of TLS-associated immune cells (T cells, B cells and DC), detailed results of which were shown in sFig 3. High expression of TLS signature was only demonstrated significant correlation with favorable survival in certain tumor types including HNSC, OV and SKCM. Tumors with high TLS scoring also exhibited tendency of improved survival in BRCA, LIHC, UCEC, although the association of which failed to achieve statistical significance. Unexpectedly, high expression of TLS signature also predicted worse outcome in patients with KIRP and KIRC (sFig 3). The prognostic relevance of TLS scoring was basically in line with that of the infiltrating level of TLS associated immune cells like T cells, B cells, cytotoxic cells and DC, although TLS signature may not necessary outperform them in prognostic prediction. We further classified the samples of each cancer types into three groups (low TLS, intermediate TLS and high TLS) according to TLS scoring, based on which cox regression survival analysis and Kaplan–Meier survival analysis was performed (Fig. 5). Similarly, tumor with high TLS exhibited significant improved overall survival in LIHC (*P* = 0.051, Fig. 5b), OV (*P* = 0.00053, Fig. 5c), SKCM (*P* = 0.027, Fig. 5d) and UCEC (*P* = 0.012, Fig. 5e). As indicated in published literature^22^, TCGA endometrial tumors are composed of four different molecular subtypes which have different survival profiles. We further evaluated the confounding effect of the molecular subtypes on the prognostic relevance of TLS. When accounting for subtype, survival differences between TLS low and high tumors was remained only in CN high subtype (sFig 5c, *P* = 0.038) but not the other subtypes (sFig 5d,e).

To further evaluate the prognostic significance of TLS density in normal tissue adjacent to the tumor, similar analysis approach was performed on the cases with available adjacent normal tissue. As shown in sFig 4, high expression of TLS signature in adjacent normal tissue was associated with unfavorable survival in BLCA (*P* = 0.0762) and LIHC (*P* = 0.0525). No obvious prognostic correlation was observed for TLS in adjacent normal tissue of the other tumor types. We failed to draw the conclusion for ACC, CESC, SKCM, GBM and LGG due to the lack of data regarding adjacent normal tissue for these tumors.

### Correlation between TLS and therapeutic response to immunotherapy

**Figure 6:**
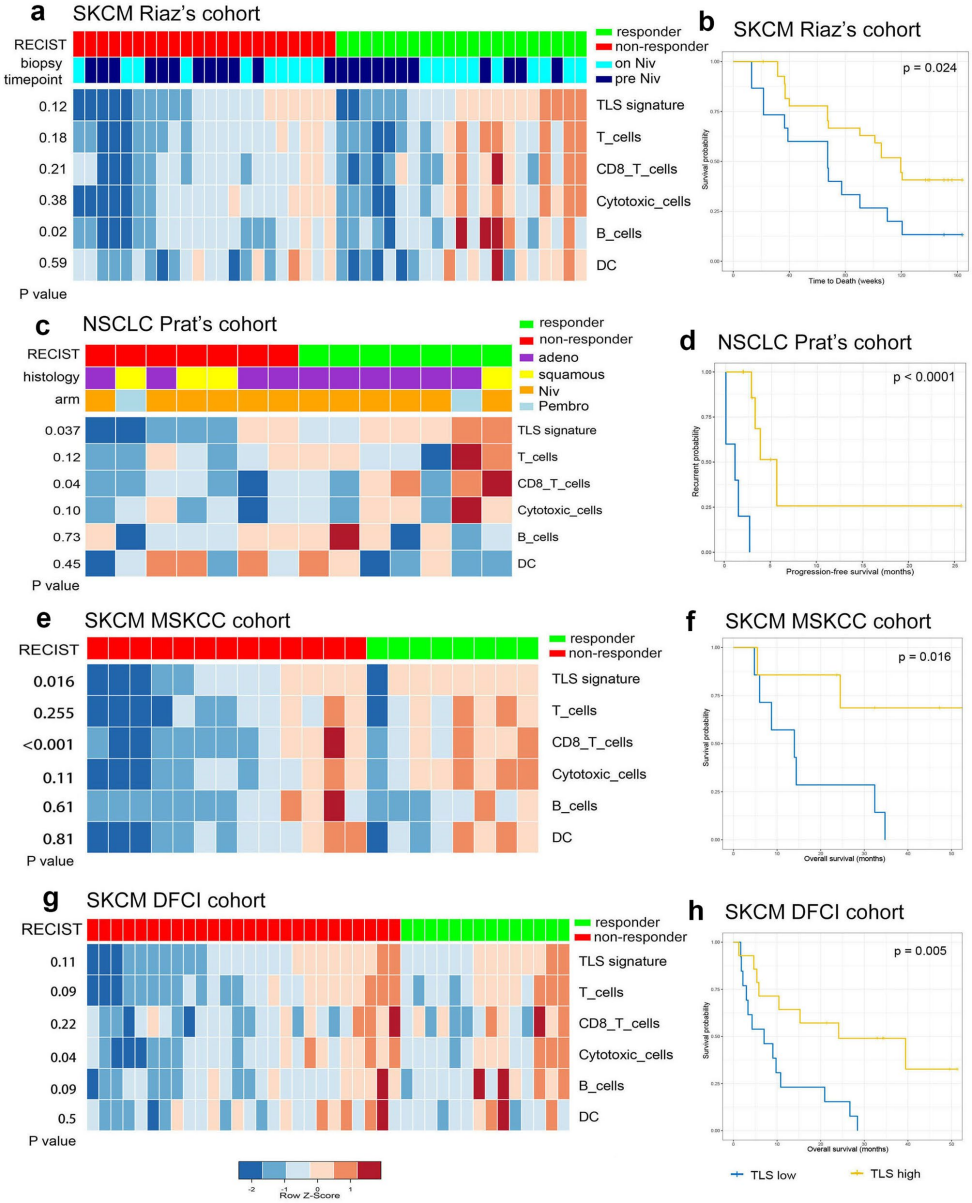
Transcriptome analysis of tumor specimens from patients with advanced NSCLC or melanoma treated with anti-PD-1 blockade. (**a**) Supervised clustering of SKCM Riaz’s cohort by response to immunotherapy (Nivolumab) (n = 22 non-responders and 21 responders), displaying enrichment level of TLS and immune cells including T cells, CD8+ T cells, cytotoxic cells, B cells and DC. *p* value for the comparison between responders and non-responders were shown. The biopsy timepoint of the specimens include pre-Niv (before Nivolumab treatment) and on-Niv (during Nivolumab treatment). (**c**) Supervised clustering of NSCLC Prat’s cohort by response to immunotherapy (Nivolumab/Pembrolizumab) (n = 7 non-responders and 7 responders), displaying enrichment level of TLS and immune cells including T cells, CD8+ T cells, cytotoxic cells, B cells and DC. *p* value for the comparison between responders and non-responders were shown; Niv, Nivolumab; Pembro, Pembrolizumab; adeno, adenocarcinoma; squamous, squamous cell carcinoma. (**e**) and (**g**) supervised clustering of SKCM MSKCC cohort (n = 13 non-responders and 8 responders) and SKCM DFCI cohort (n = 26 non-responders and 14 responders) by response to anti-CTLA-4 immunotherapy (ipilimumab), displaying enrichment level of TLS and immune cells including T cells, CD8+ T cells, cytotoxic cells, B cells and DC. *p* value for the comparison between responders and non-responders were shown. Kaplan–Meier plots demonstrating the prognostic difference between patients with high TLS scoring (> the second tertile) and low TLS scoring (< the second tertile) in SKCM Riaz’s cohort (**b**), SKCM MSKCC cohort (**f**), SKCM DFCI cohort (**h**) in terms of overall survival, and in NSCLC Prat’s cohort (**d**) in terms of progression-free survivals.

Utilizing the transcriptome and clinical data derived from published studies of immunotherapy clinical trials, we were able to verified the predictive value of TLS signature in the therapeutic efficacy of immunotherapy. Two cohorts (SKCM Riaz’s cohort and NSCLC Prat’s cohort) of anti-PD-1 therapy involving patients diagnosed with advanced NSCLC and SKCM respectively were included in the present study. All the patients had failed the primary line of treatment but were immunotherapy naïve before recruited to clinical trial of anti-PD-1 immunotherapy (nivolumab or pembrolizumab). As shown in Fig. 6a, SKCM patients responding to anti-PD-1 blockade demonstrated higher expression of TLS signature and infiltrating level of TLS-associated immune populations like T cells, B cells, CD8 T cell, cytotoxic cells and DC as compared to non-responder, although the difference was not statistically significant. Patients with high TLS scoring also demonstrated significant improved overall survival (*P* = 0.024) (Fig. 6b). Similar findings were also observed in NSCLC dataset. Specifically, TLS signature was significantly enriched in patients responding to immunotherapy (*P* = 0.037) (Fig. 6c). Also, NSCLC patients with high TLS scoring were associated with significantly prolonged progression-free survival (*P* < 0.0001) (Fig. 6d). Therapeutic relevance of TLS scoring in anti-CTLA-4 immunotherapy were also evaluated in two cohorts (SKCM MSKCC cohort and SKCM DFCI cohort) involving cases of advanced SKCM. Similarly, cases with high TLS scoring and infiltrating level of TLS-associated immune populations (T cells, B cells, CD8 T cell, cytotoxic cells and DC) were predominantly observed among responders (Fig. 6e, g). High TLS scoring significantly correlated with favorable overall survival (*P* < 0.05) (Fig. 6f, h). These results indicated that enrichment score of TLS signature can serve as a promising prognostic biomarker in immunotherapy.

## Discussion

Based on the publicly available large-scale genomic data sets of solid tissue tumors and the corresponding clinical information, we quantified TLS density in each sample utilizing the previously defined 12-chemokine signature and thoroughly evaluated its correlation with tumor genomic features and prognosis in pan-cancer. Our findings suggested that neoantigen load, oncovirus infection and diver gene mutations may play a role in the formation of TLS, which in turn also interact with tumor cells and help to shape the oncogene mutation landscape across different tumor types. High expression of TLS signature signify favorable prognosis in certain cancer types and robustly predicted the response to immunotherapy in melanoma and NSCLC.

Differing from the previous work that characterized TLS based on the presence of TLS specific immune cells like DC-Lamp+ mature DC or CD20+ follicular B cells etc. on IHC^7,23^, we quantified the density of TLS based on a molecular immune signature derived from the immune gene array profiling of ectopic lymph node-like structures in CRC^18^. Instead of physical capture the presence of TLS, the signature based approach characterized TLS from the perspective of its functional status as evidenced by the expression of TLS specific chemokines, the robustness of which had been verified in multiple studies involving lung cancer, breast cancer, melanoma, LIHC etc.^10,11,20,21^. As shown in our results, expression of the 12-chemokine signature demonstrated the most strong correlation with immune cells including B cells, T cells, Th1, CD8+ T cells, DC, macrophages etc., which had been identified as the major component of TLS^3,24,25^. These findings also support the feasibility of TLS quantification based on the 12-chemokine signature.

As decided by the enrichment score of the gene signature, TLS density in different tumor types differ dramatically with one another. As expected, the lowest TLS scoring was observed in LGG, GBM ect., which were all well known as immune cold tumor types with scarce immune cell infiltration^26,27^. Among the top 6 tumor types that highly expressed TLS signature, LUAD, LUSC, and SKCM were also the tumor that achieve the best response to checkpoint blockade^28–31^, indicating the potential involvement of TLS in the anti-tumor response during immunotherapy. Higher TLS scoring was found in tumor tissue as compared to adjacent normal tissue for most cancer types, which is consistent with the notion that TLS is mainly induced at sites where chronic inflammation persists^3^. The only exception is LIHC where TLS scoring in adjacent normal tissue is higher than that of tumor tissue, which might be attributed to the preexisted abundant immune cells in the liver as an immune organ^32^. We didn’t observe an ubiquitous correlation between tumor staging and TLS abundance, hinting the alteration of TLS along with tumor progression is non-significant or cancer-type dependent.

Virus infection had been linked with the process of carcinogenesis in multiple tumor types, like HBV infection in the development of hepatocellular carcinoma, HPV in the oncogenesis of cervical carcinoma etc.^33–35^. Chronic viral infection is also capable of eliciting the formation TLS at the site of infection^36,37^. We first want to asked whether the presence of viral infection during oncogenesis play a role in the formation of TLS within TME. For some tumor types, we did observed that the presence of viruses significantly associated with increased expression of TLS signature. Specifically, positive results were observed for EBV infection in STAD and HPV infection in HNSC. Our findings indicated that certain virus infection may trigger the formation of TLS within TME, although the effect might be cancer-type dependent.

The positive correlation between cytotoxic T cell infiltration and neoantigen abundance within TME had been well characterized in previous studies^38,39^. As the primary site of antigen presentation within TME, TLS bridge the connection between tumor-derived neoantigens and production of anti-tumor effector T cells. While the positive correlation of TLS density with the cytotoxic immune contexture as well as the T cell receptor repertoire clonality had been clearly evidenced in previous studies^40,41^, the association between TLS abundance and neoantigen load had never been evaluated. We found that neoantigen load inferred from the genomic mutation data were positively correlated with TLS scoring in multiple cancer types, so was the total mutation burden. Also, tumor types with high mutation/neoantigen load tend to have higher expression of TLS signature. Our finding support the speculation that high tumor-derived neoantigen burden play a role in triggering the formation of TLS in TME.

Certain oncogenic events caused by driver gene mutation like CTNNB1^42^, FGFR3^43^ etc. had been associated with tumor intrinsic immune exclusion. Thus, we want to further evaluate whether mutation on certain driver genes would interfere with TLS formation, or in turn, whether TLS formation would alter the mutational landscape that facilitate immune escape. Consistent with the published finding, we found that gain-of-mutation in CTNNB1 associated with decreased TLS scoring in the overall solid tumors. Several tumor suppressor genes like CASP8, KEAP1, PBRM1, FBXW7 etc. were found to be associated with increased expression of TLS signature when harboring loss-of-function mutation. It can be interpreted as an adaptation acquired by the tumor cells in response to the stress posed by immune response, when only tumor with high oncogenesis or aggressiveness can survive. Of note, a recent study had demonstrated the involvement of caspase-8 (encoded by CASP8) in the cleavage of immune regulator, RIPK1, the accumulation of which can cause autoinflammatory diseases^44^. Thus, overexpression of TLS signature in tumor with genomic loss in CASP8 could also be associated with its immune dysregulation function. Tumors with high TLS scoring were also frequently correlated with loss of function mutation on genes associated with antigen presentation, like HLA-A, HLA-B, or genes involved in immune regulation like SMAD4^45^, which could be the mechanism by which tumor cells escape the immune surveillance. Intriguingly, several genes associated with DNA repair, like BRCA1^46^, BRCA2^46^ and TP53BP1^47^, were also frequently mutated (loss-of-function) in tumors with high TLS scoring. As shown previously, neoantigen load and mutation frequency were positively correlated with TLS scoring. We speculated that high TLS density in tumors with mutated BRCA1, BRCA2 or TP53BP1 could be attributed to the genomic instability and the subsequent increase in mutation burden. All these findings indicate the interaction between TLS in TME and the oncogenic alteration within tumor cells, although detailed mechanism by which they exert an effect on each other remains to be elucidated by further study.

Quite a few studies involving different solid tumors had sought to evaluate the prognostic and predictive impact of TLS within tumor mass, the yielded results remains in dispute^7,9,48–50^. The prognostic significance of TLS in the peri-tumor tissue was also controversial, with some studies claiming it to be prognostic favorable^51,52^, others reporting it to be prognosis irrelevant^11^, and still others suggesting it heralds adverse outcome^10,53^. In the present study, we thoroughly evaluate the prognostic impact of TLS in the tumor core or the adjacent normal tissue for patients after the surgical removal of tumors. We found that high expression of TLS signature in tumor tissue signify favorable outcome for only certain tumor types including SKCM, OV, LIHC and UCEC. We also detailly evaluated the confounding effect of molecular subtypes of UCEC, and found that survival relevance of TLS was less significant when accounting specific molecular subtypes. We also found that tumor subtypes with copy number alteration (CN low or CN high) demonstrated significantly lower TLS scoring as compared to MSI (hypermutated) subtype and POLE (ultramutated) subtype. It’s consistent with the previous study that highlighted the negative correlation between somatic copy number alteration and immune inflation^54^. As the molecular subtypes of endometrial cancer were characterized based on genomic alteration, it’s not a surprise that the molecular subtypes would confound the TLS related analysis. We didn’t observe any survival benefit in lung cancers with high TLS scoring, which though had been reported in published studies^7^. The inconsistent findings could be attributed to the difference in the methods applied for TLS quantification. TLS in the adjacent normal tissue demonstrated the tendency of signifying adverse outcome especially in LIHC and BLCA, although none of them achieved statistical significance, which might be associated with the modest sample size.

Recent evidences from the clinical trials had emphasized the clinical significance of TLS in predicting response to neoadjuvant immunotherapy in patients with NSCLC and melanoma^14,15^. Yet no study had evaluated predictive value of TLS in therapeutic efficacy of immunotherapy as the second or third line treatment. To address this issue, we made used of the published data regarding immunotherapy in patients with advanced NSCLC or melanoma. We successfully proved the robustness of TLS signature in predicting therapeutic response or survival outcome in patients receiving immunotherapy after fail the primary line of treatment. Our results further verified that TLS density in tumor tissue can guide patients stratification for immunotherapy.

Our current work is the first study that thoroughly evaluated the molecular features and association with TLS and its prognostic or predictive significance in pan-caner. Though the limitations of our study need to be clearly addressed. First of all, the current work was merely based on the in silico analysis of TCGA, with TLS density and all the other immune parameters inferred from the transcriptome data. All these findings need to be confirmed by further study integrating pathological data. As for the analysis regarding driver gene mutation, we only included genes demonstrated statistical significance in pan-cancer analysis, which might neglect genes significantly associated with TLS in selected tumor types but not in the pan-cancer setting. Also, we failed to verified these findings especially with regard to the correlation between driver gene mutation and TLS in another pan-cancer dataset due to the limited resource. Thirdly, the predictive value of TLS in immunotherapy was only verified in limited datasets with modest sample size. Further clinical studies is warranted to verify its clinical significance in guiding immunotherapy.

In conclusion, the present study delineated the association between TLS formation in TME and the molecular features like oncovirus infection, neoantigen burden and driver gene mutation landscape. TLS convey significant prognostic and predictive value in surgical treatment or immunotherapy of different solid tumors. This findings brings insight into the mechanism and significance of TLS formation, which can be utilized to maximize therapeutic benefit in the era of immunotherapy.

## Materials and methods

### Data sets of tumor and normal samples

The Cancer Genome Atlas (TCGA) is a landmark cancer genomics program that molecularly characterized more than 20,000 tumor samples and matched normal samples spanning 33 cancer types, whose data were publicly available and facilitate all kinds of cancer research. Fastq files of RNA-Seq data for 8672 tumor samples and 741 normal samples across 24 cancer types downloaded from TCGA hub (https://portal.gdc.cancer.gov/) were preprocessed with "Rsubread" R package, which included aligning the reads according to the UCSC hg19 reference genome, summarizing the gene level expression values as integer number, and normalizing the expression level to FPKM and TPM values. The reprocessed RNA-Seq data and corresponding clinical data of each tumor were stored in gene expression omnibus (GEO) as GSE62944 dataset, which is freely available to the public. As our study only focus on solid tumors, RNA-seq data (FPKM) of 22 types of solid tumors and the corresponding adjacent normal tissue were downloaded from GEO database (GEO: GSE62944). Clinical information including histological diagnosis, pathological staging and survival data (overall survival time and survival status) were also collected from GSE62944 for each samples. TCGA melanoma (SKCM) cohort contained three quarters metastatic tumors, the majority of these are from lymph nodes. To get rid of the confounding effect of metastatic tumors, we only included melanoma of primary sites (n = 106) in the current study. A total of 8672 tumor samples and 619 adjacent normal samples were included in the present study (more detailed information were shown in supplementary Table S1).

### Data obtaining from immunotherapy clinical trial

In order to verify the relationship between TLS density and clinical response to immunotherapy, published data of four cohorts from immunotherapy trials (SKCM Riaz’s cohort^55^, NSCLC Prat’s cohort^56^, SKCM MSKCC cohort^57^ and SKCM DFCI cohort^58^) with publicly available clinical and transcriptome data were obtained and reanalyzed in the present study. Both SKCM Riaz’s cohort and NSCLC Prat’s cohort were derived from clinical trials of anti-PD-1 immunotherapy, whereas SKCM MSKCC cohort and SKCM DFCI cohort all received anti-CTLA4 (ipilimumab) treatment. A total of 43 ipilimumab-naïve patients with advanced melanoma underwent PD-1 blockage (Nivolumab) from Riaz’s study^55^ were included in the present study to constituted SKCM Riaz’s cohort. NSCLC Prat’s cohort involved 14 patients with advanced non-small cell lung cancer (NSCLC) underwent PD-1 (Programmed cell death protein 1) blockage treatment (Pembrolizumab or Nivolumab) in Prat’s study^56^, where only patients with archival samples that obtained prior to any medical treatment were recruited in the current study. Transcriptome data of Riaz’s cohort and Prat’s cohort were downloaded from GEO database (GSE93157, GSE91061), while clinical information were obtained from the supplementary material of corresponding studies. Clinical and transcriptome data of SKCM MSKCC cohort involving 21 cases and SKCM DFCI cohort involving 40 cases were all obtained from cBioportal database (https://www.cbioportal.org/). For the convenience of analysis, patients with complete response, partial response and stable disease as decided by Response Evaluation Criteria in Solid Tumors (RECIST) after immunotherapy were all categorized as response group, while patients with progress disease were categorized as non-response group. Detailed information of the four cohorts were shown in supplementary Tables S2–5.

### Estimation of TLS and immune cell enrichment from transcriptome data

A metagene comprising 12 Chemokines (CCL2, CCL3, CCL4, CCL5, CCL8, CCL18, CCL19, CCL21, CXCL9, CXCL10, CXCL11, and CXCL13) that highly expressed by TLS was applied as the gene signature of TLS. Based on the whole exome transcriptome data, enrichment score of TLS was calculated by single-sample gene set enrichment analysis (ssGSEA)^59^ method as implemented by R-package (GSEABase and GSVA). Detailed information regarding the TLS score for each TCGA samples had been listed on supplementary Table S6.

xCell is a webtool that performs cell type enrichment analysis for different immune and stroma cell types based on the bulk-tissue RNA-seq data by integrating single-sample gene set enrichment analysis (ssGSEA) and deconvolution methods^60^. The abundance of 24 types of immune cell or stromal components in tumor mass was estimated by xCell using Bindea signatures (https://xcell.ucsf.edu/). Transcriptome data were processed by xCell to generate enrichment scores for each immune cell type across all samples.

### Identification of viral infection based on RNA-seq data

The evaluation of virus infection across tumors was retrieved from previous publications^39,61^. Basically, unaligned (non-human) reads were from TCGA RNA-seq data in BAM format were extracted after filtering of human content, and were aligned to the reference sequence collection of viral genome to identify the presence of viral infection^61^. Positive identification required at least 300 nt of unique sequence to map to the viral genome and the expression of a virus was higher than the observed across healthy tissues^39^.

### Quantification of mutation/neoantigens load

Mutation counts and predicted neoantigens of each samples were retrieved from previous publication^39^. As described in previous literature, somatic mutations were annotated with VEP (Variant Effect Predictor), and non-synonymousn mutations in protein coding regions were counted as the tumor mutational burden (TMB)^38^. To predict whether a mutation yield neo-epitopes or not, affinity of the novel amino acid produced by the mutated genes binding to the patient’s germline HLA alleles was decided by a previously developed algorithm NetMHCpan (v2.4)^62,63^.

### Acquisition of driver gene mutations

A total of 375 candidate genes were identified as the potential driver mutations in the previous study by applying MutSigCV to exome sequences from large-scale tumor–normal pairs samples^64^. Mutation status of all the candidate genes were obtained from cBioportal database (https://www.cbioportal.org/). Specifically, we utilized the Firehose Legacy dataset (formerly Provisional datasets) on cBioportal, whose original mutation data for each TCGA cancer type were obtain from the Broad Firehose and processed a legacy processing pipeline. Only genes with functional annotated mutations (loss-of-function mutation, gain-of-function mutation, switch-of-function mutation) were applied to further analysis. Samples with mutation of unknown significance on a gene were excluded from the analysis regarding this specific gene. We also excluded genes where both loss-of-function and gain-of-function mutations exist.

### Statistical analysis

Assessments of difference in continuous variables between two groups were decided by two-sample t test. Specifically, pair-wise t-test was performed in the comparison of TLS scoring between two tumor samples and normal tissue. Pearson correlation analysis was carried out to determine the correlation between TLS abundance and infiltration of different immune cell population across all the tumor types. Spearman rank correlation analysis was performed to evaluate the correlation between TLS abundance and mutation/neo-antigens load. Candidate genes were tested for the association between functional mutation and TLS enrichment score using binomial logistic regression analysis with TLS scoring as the independent variable, mutational status of gene in question as the dependent variables, and *P* < 0.01 as the threshold for identification of genes in statistical significance in the pan-cancer setting. Genes selected in pan-cancer analysis were further applied to analysis in each tumor types by the same approach. Survival correlation of TLS scoring and infiltrating level of certain immune cells as continuous variable were tested by univariate cox regression survival analysis. For each tumor types, TLS scoring were also transferred into ordinal categorical variable (low, the first tertile; intermediate, the second tertile; high, the third tertile) and were applied to the cox proportional hazards regression analysis for each tumor types. Kaplan–Meier plots (log-rank test) was also performed for selected tumor types to compare the prognosis between tumors with low and high TLS scoring. Overall survival times were defined as months from initial pathological diagnosis to death or the last time the patient was known to be alive. Progression free survival times were defined as weeks from the initiation of treatment to the first identification of progression or the last time of follow-up. All statistical analyses and data presentations were performed in R language 3.6.1 (http://www.r-project.org).

## Supplementary information

Supplementary Figure Legends.

Supplementary Figure 1.

Supplementary Figure 2.

Supplementary Figure 3.

Supplementary Figure 4.

Supplementary Figure 5.

Supplementary Table S1.

Supplementary Table S2.

Supplementary Table S3.

Supplementary Table S4.

Supplementary Table S5.

Supplementary Table S6.
