# Supplementary material for: Pan-cancer analysis of genomic properties and clinical outcome associated with tumor tertiary lymphoid structure
Source: Sci Rep. 2020 Dec 9;10:21530. doi: 10.1038/s41598-020-78560-3 (PMC7725838; doi:10.1038/s41598-020-78560-3)
Supplement: Supplementary file 1 — Supplementary Figure Legends. [file 41598_2020_78560_MOESM1_ESM.docx]

# Supplementary figure legends

**Fig 1. Pan-cancer analysis of mutation counts and predicted neoantigen load.**

(a) Scatter plot demonstrating the correlation between mutation counts and neoantigen load in pan-cancer, with dots of different colors representing different tumor types; R value and P value of Pearson correlation analysis are shown. (b) Boxplot demonstrating mutation counts across 18 tumor types; (c) Boxplot demonstrating predicted neoantigen counts across 18 tumor types.

**Fig 2. Correlation between TLS scoring and mutation/neoantigen load across different solid tumor types**

Scatter plot with local regression curves showing the relationships of TLS scoring with total mutation count and predicted neoantigen load in different solid tumor types; R value, P value and false discovery rate (FDR) of Spearman rank correlation analysis of each tumor type were presented; Curves span the 5th to 95th percentile of the mutation/neoantigen counts variable.

**Fig 3. Prognostic significance of TLS scoring and infiltration of certain immune cells across different solid tumors.**

Univariate cox regression survival analysis was performed for TLS scoring and infiltration of B cells, T cells, CD8 T cells, cytotoxic cells, DC as continuous variables across different tumor types, P value for each variables in each tumor type was shown. Parameters of favorable prognostic significance was denoted in green, and parameters of unfavorable prognostic significance was denoted in red. Parameters with significant prognostic significance (P<0.05) were shown with border.

**Fig 4. Prognostic impact of TLS scoring in adjacent normal tissue of different solid tumors.**

Forest plot demonstrated the survival impact of TLS level in adjacent normal tissue of each tumor type. Specimens were categorized into high TLS scoring (>the second tertile), intermediate (between the first and the second tertile) TLS scoring and low TLS scoring (<the first tertile) for adjacent normal samples of each tumor types. Hazard Ratio [5%-95% confidence interval] and P value were obtain by cox regression survival analysis.

**Fig 5. Prognostic impact of TLS in UCEC accounting for molecular subtypes.**

(a) Kaplan-Meier plots of overall survival difference between UCEC of different molecular subtypes; (b) boxplot demonstrating TLS scores of different molecular subtypes; (c) Kaplan-Meier plots of overall survival difference between tumors with high TLS scoring and low TLS scoring in CN high subtype; (d) Kaplan-Meier plots of overall survival difference between tumors with high TLS scoring and low TLS scoring in CN low subtype; (e) Kaplan-Meier plots of overall survival difference between tumors with high TLS scoring and low TLS scoring in MSI subtype. TLS high, tumors with TLS scoring > the second tertile; TLS high, tumors with TLS scoring < the first tertile.
