# Supplementary figures and images for: Pan-cancer analysis of genomic properties and clinical outcome associated with tumor tertiary lymphoid structure
Source: Sci Rep. 2020 Dec 9;10:21530. doi: 10.1038/s41598-020-78560-3 (PMC7725838; doi:10.1038/s41598-020-78560-3)

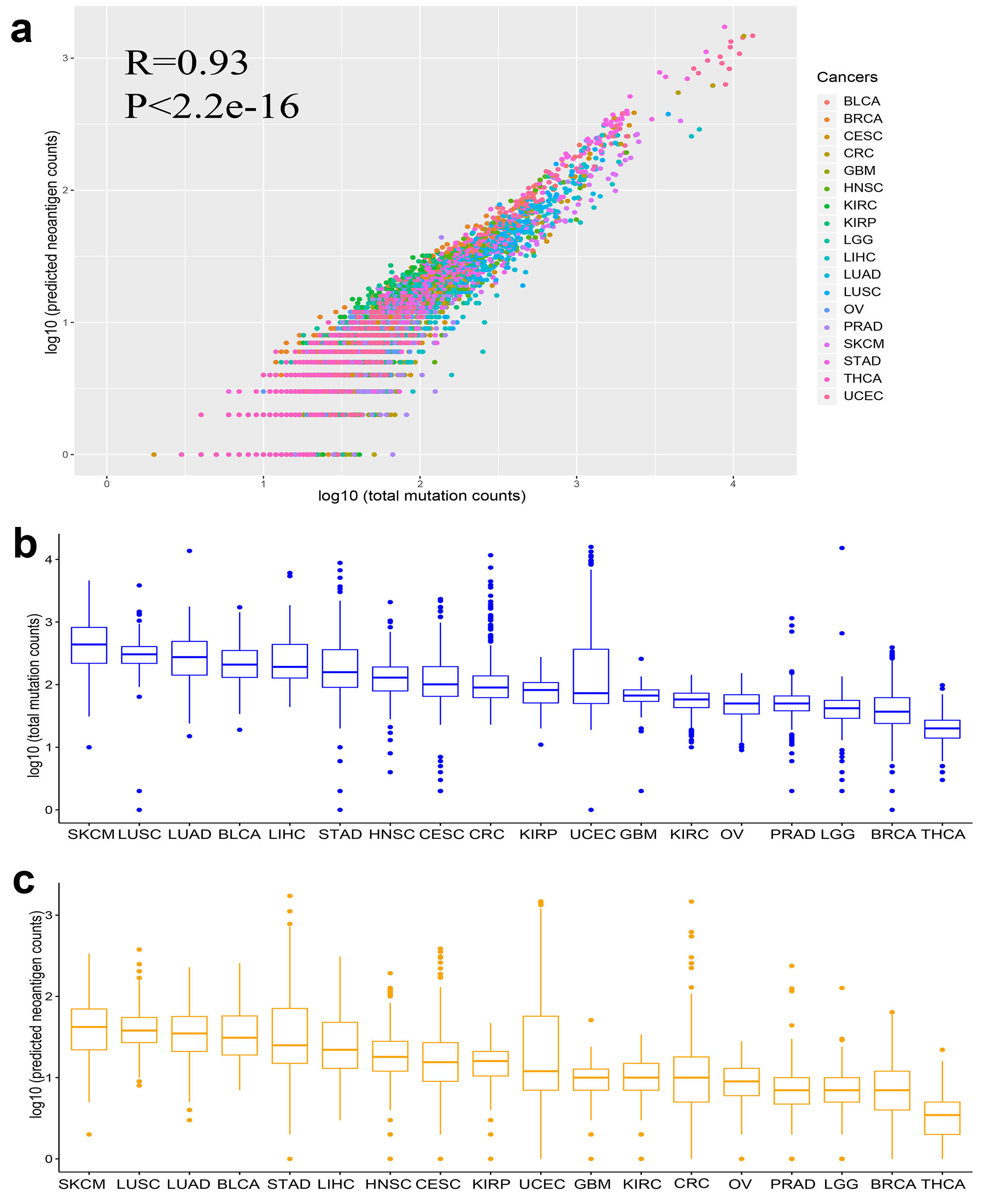

Supplement: Supplementary file 2 — Supplementary Figure 1. [file 41598_2020_78560_MOESM2_ESM.jpg]

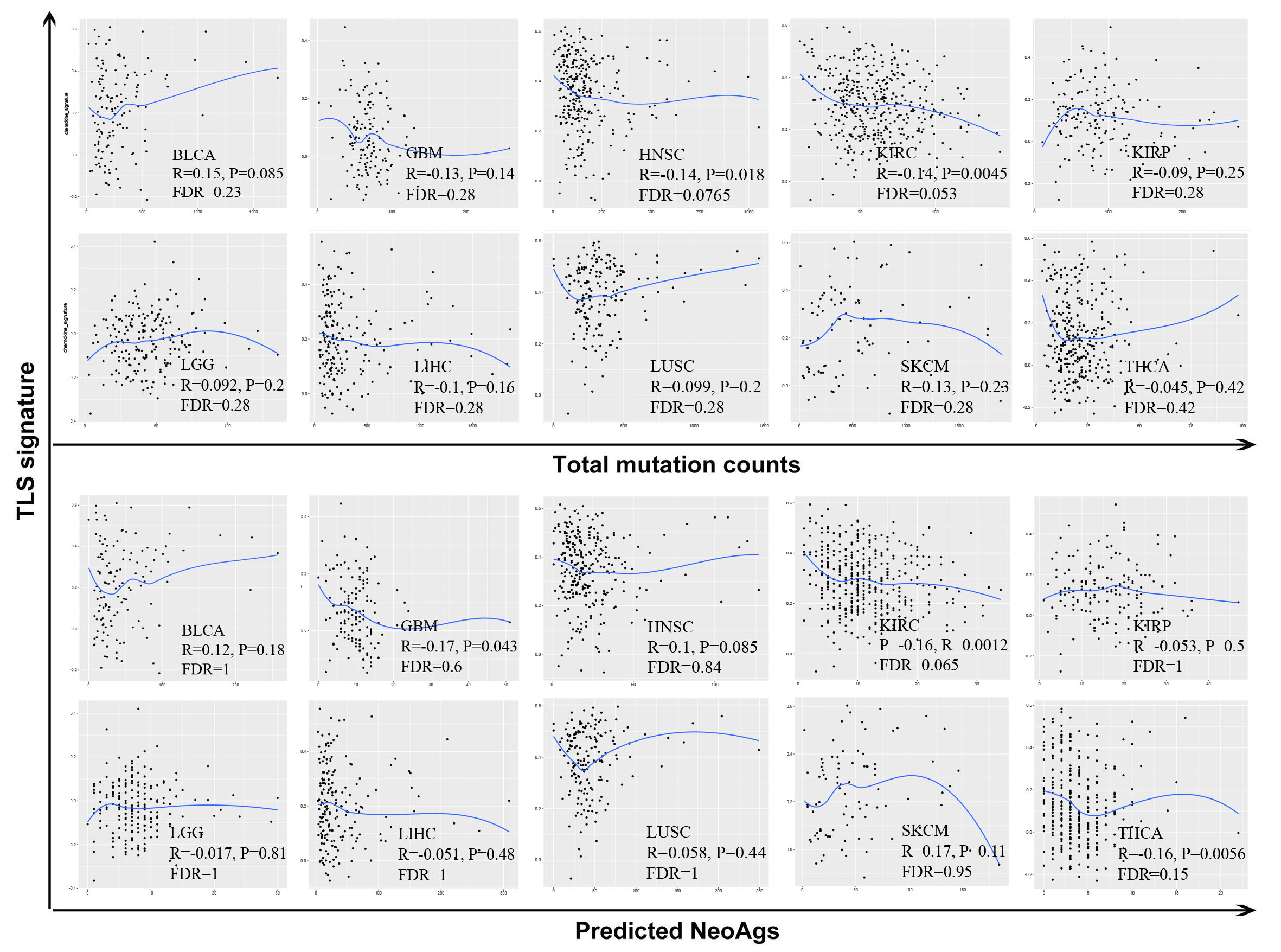

Supplement: Supplementary file 3 — Supplementary Figure 2. [file 41598_2020_78560_MOESM3_ESM.jpg]

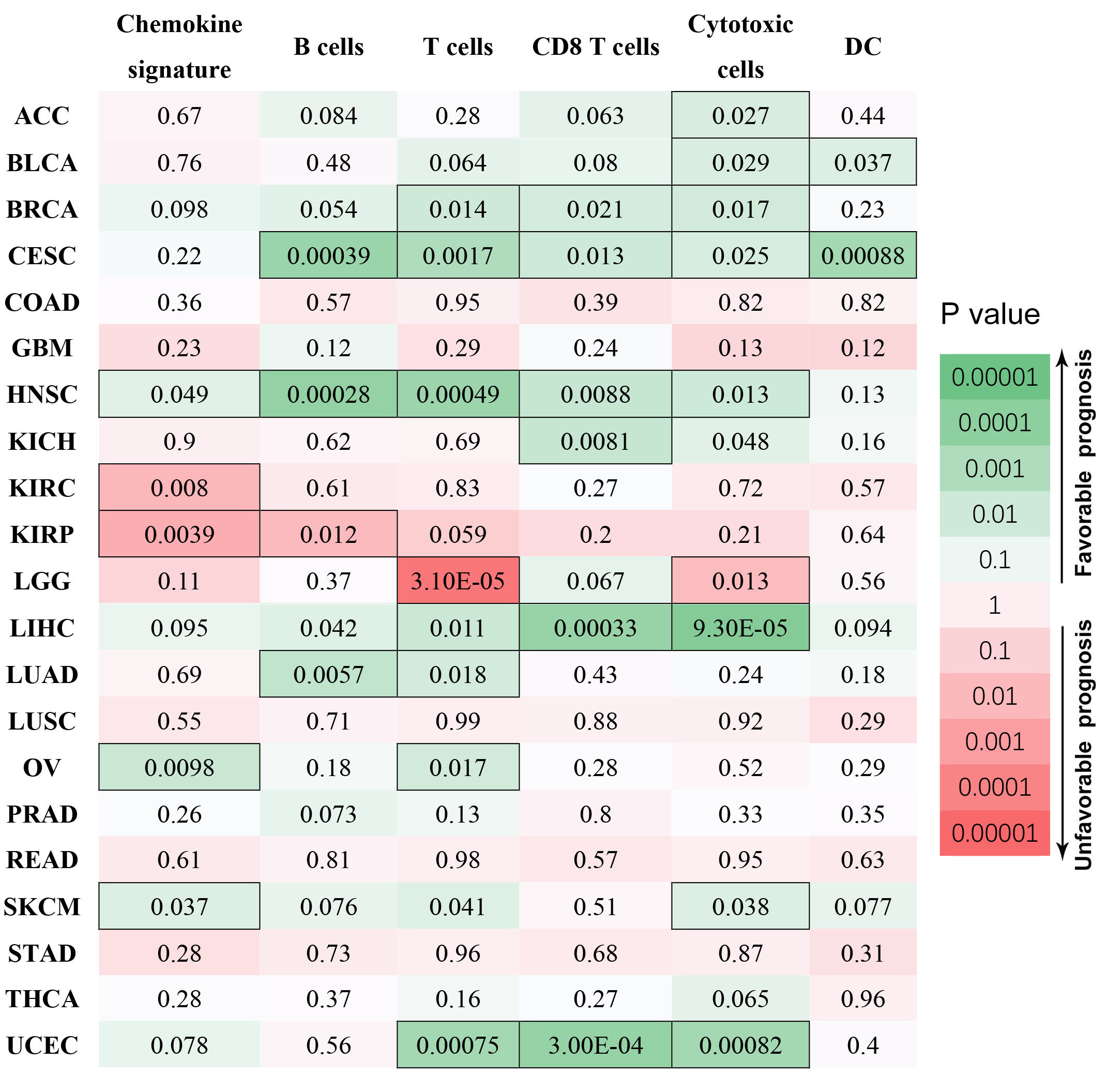

Supplement: Supplementary file 4 — Supplementary Figure 3. [file 41598_2020_78560_MOESM4_ESM.jpg]

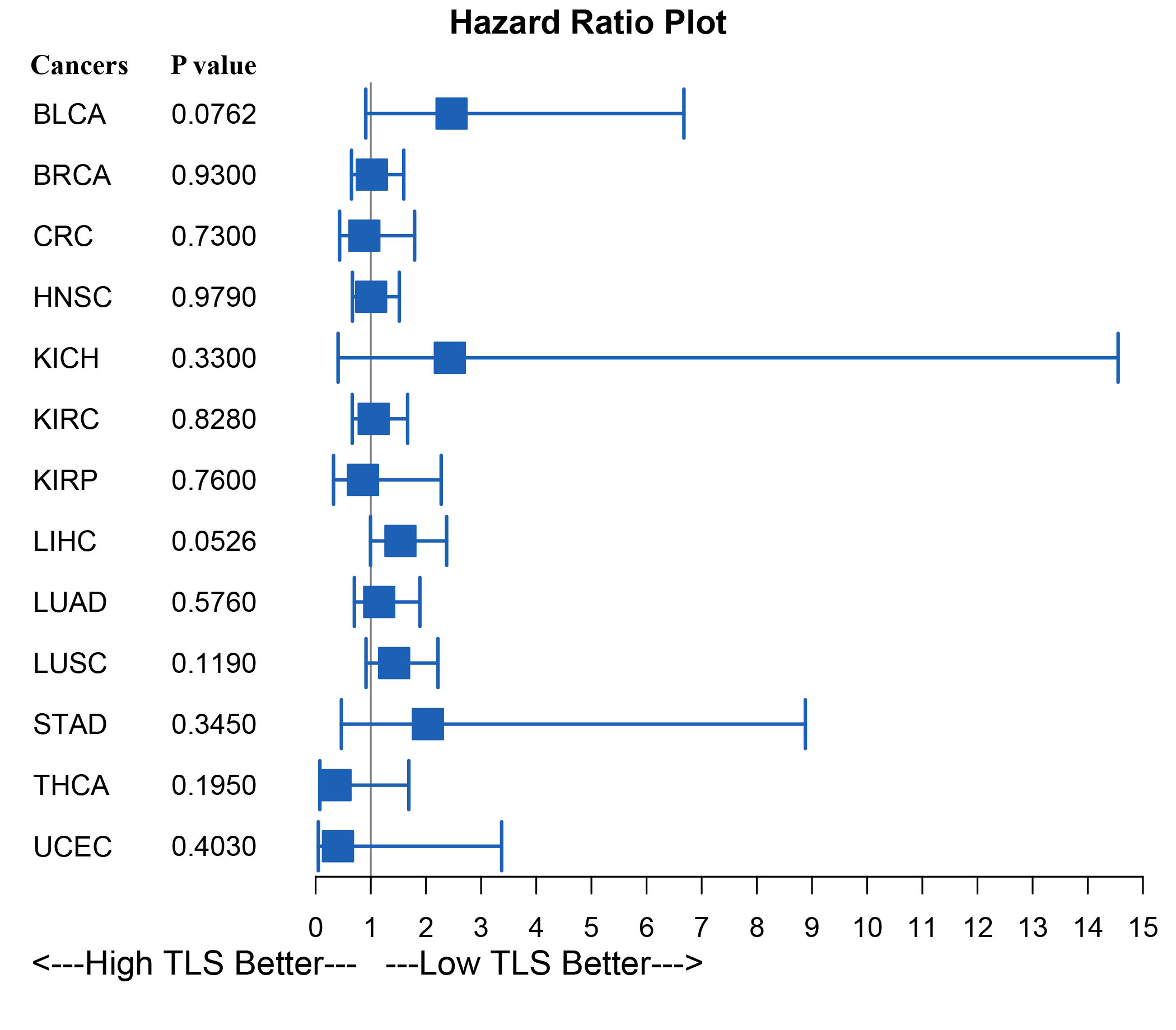

Supplement: Supplementary file 5 — Supplementary Figure 4. [file 41598_2020_78560_MOESM5_ESM.jpg]

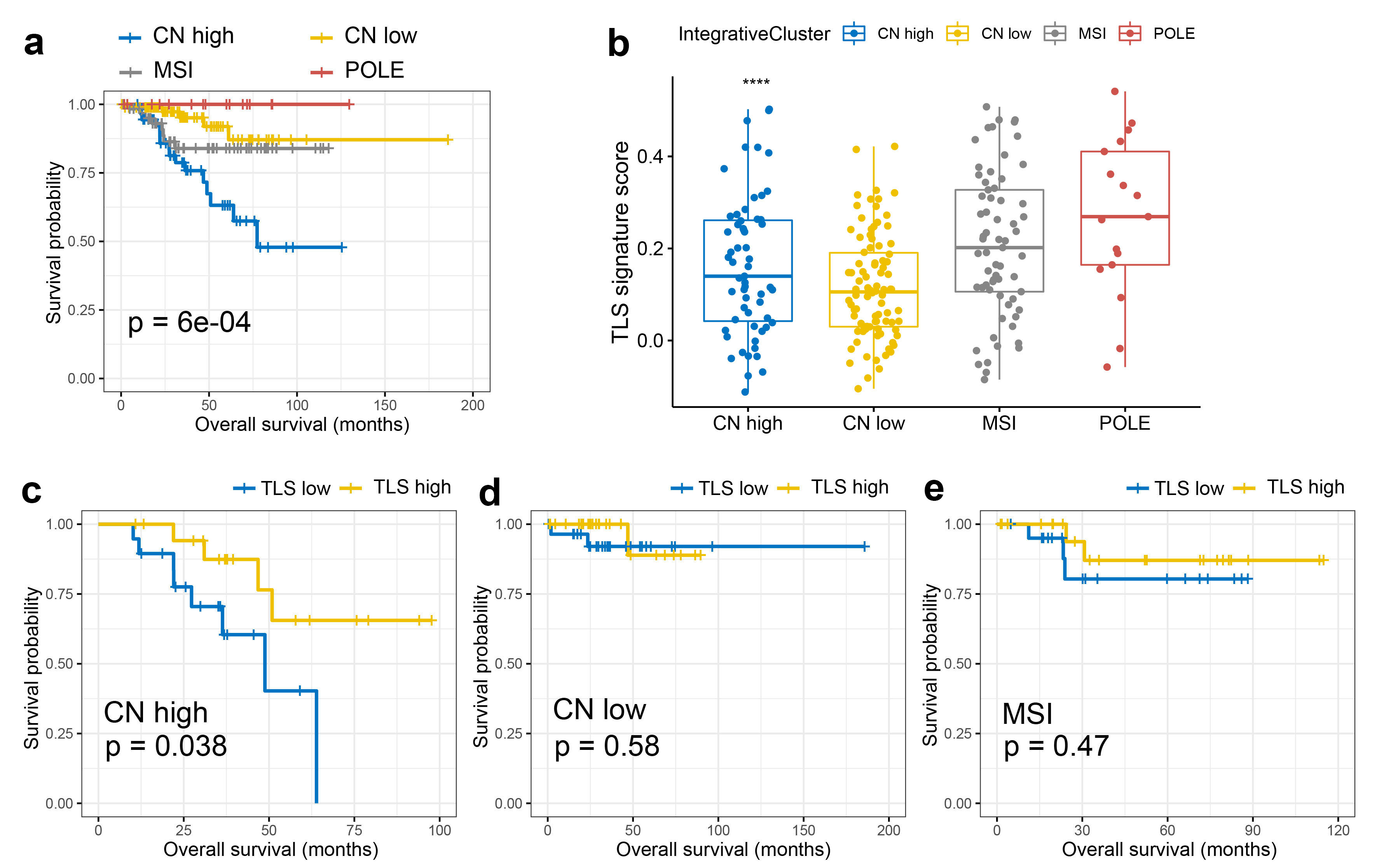

Supplement: Supplementary file 6 — Supplementary Figure 5. [file 41598_2020_78560_MOESM6_ESM.jpg]
