# Supplementary material for: Pan-cancer analysis of genomic properties and clinical outcome associated with tumor tertiary lymphoid structure
Source: Sci Rep. 2020 Dec 9;10:21530. doi: 10.1038/s41598-020-78560-3 (PMC7725838; doi:10.1038/s41598-020-78560-3)
Supplement: Supplementary file 7 — Supplementary Table S1. [file 41598_2020_78560_MOESM7_ESM.docx]

**Supplementary Table S1. Summary of the basic information of 22 solid tumors**

| Cancer | Name | Tumor specimens | Peri-tumor specimens |
| --- | --- | --- | --- |
| ACC | Adrenocortical carcinoma | 79 | 0 |
| BLCA | Bladder Urothelial Carcinoma | 414 | 18 |
| BRCA | Breast invasive carcinoma | 1119 | 93 |
| CESC | Cervical squamous cell carcinoma and endocervical adenocarcinoma | 306 | 0 |
| COAD | Colon adenocarcinoma | 483 | 41* |
| GBM | Glioblastoma multiforme | 170 | 0 |
| HNSC | Head and Neck squamous cell carcinoma | 504 | 43 |
| KICH | Kidney Chromophobe | 66 | 24 |
| KIRC | Kidney renal clear cell carcinoma | 542 | 71 |
| KIRP | Kidney renal papillary cell carcinoma | 291 | 32 |
| LGG | Brain Lower Grade Glioma | 532 | 0 |
| LIHC | Liver hepatocellular carcinoma | 374 | 50 |
| LUAD | Lung adenocarcinoma | 541 | 50 |
| LUSC | Lung squamous cell carcinoma | 502 | 45 |
| OV | Ovarian serous cystadenocarcinoma | 430 | 0 |
| PRAD | Prostate adenocarcinoma | 502 | 0 |
| READ | Rectum adenocarcinoma | 167 | 41* |
| SKCM | Skin Cutaneous Melanoma | 106 | 0 |
| STAD | Stomach adenocarcinoma | 420 | 20 |
| THCA | Thyroid carcinoma | 513 | 56 |
| UCEC | Uterine Corpus Endometrial Carcinoma | 554 | 0 |
| UCS | Uterine Carcinosarcoma | 57 | 0 |
| Total |  | 8736 | 619 |

*denote the number of peri-tumor specimens in colorectal dataset (comprising COAD and READ)
