# Supplementary material for: Pan-cancer analysis of genomic properties and clinical outcome associated with tumor tertiary lymphoid structure
Source: Sci Rep. 2020 Dec 9;10:21530. doi: 10.1038/s41598-020-78560-3 (PMC7725838; doi:10.1038/s41598-020-78560-3)
Supplement: Supplementary file 8 — Supplementary Table S2. [file 41598_2020_78560_MOESM8_ESM.docx]

**Supplementary Table S2. Clinical information of NSCLC patients in Prat. Dataset**

| patient ID | histology | PREV LINES | biopsy time point | arm | response | progression | PFS (months) | TLS score | B cells | T cells | CD8 T cells | | Cytotoxic cells | DC |
| --- | --- | --- | --- | --- | --- | --- | --- | --- | --- | --- | --- | --- | --- | --- |
| SAMPLE_6 | adeno | 1 | achival | Nivolumab | CR | no | 25.73 | 0.01 | -0.08 | -0.07 | -0.12 | | -0.07 | -0.06 |
| SAMPLE_7 | adeno | 3 | achival | Nivolumab | PD | yes | 1.00 | -0.29 | -0.12 | -0.11 | -0.09 | | -0.06 | -0.04 |
| SAMPLE_8 | adeno | 4 | achival | Nivolumab | PD | yes | 0.00 | -0.59 | -0.09 | -0.23 | -0.18 | | -0.11 | -0.30 |
| SAMPLE_9 | adeno | 1 | achival | Nivolumab | SD | no | 1.93 | 0.14 | 0.05 | -0.06 | -0.18 | | -0.10 | -0.08 |
| SAMPLE_10 | adeno | 2 | achival | Nivolumab | PD | yes | 2.80 | 0.63 | -0.08 | -0.12 | -0.08 | | -0.09 | -0.21 |
| SAMPLE_11 | adeno | 2 | achival | Nivolumab | SD | no | 2.87 | 0.54 | -0.25 | -0.14 | 0.00 | | -0.06 | -0.22 |
| SAMPLE_12 | adeno | 1 | achival | Pembrolizumab | SD | yes | 3.77 | 0.66 | -0.20 | 0.02 | 0.02 | | 0.05 | -0.25 |
| SAMPLE_13 | adeno | 2 | achival | Nivolumab | PR | no | 4.90 | 0.58 | -0.06 | -0.31 | | -0.16 | -0.14 | -0.13 |
| SAMPLE_14 | adeno | 1 | achival | Nivolumab | PD | yes | 3.20 | 0.59 | -0.08 | -0.18 | | -0.22 | -0.16 | -0.06 |
| SAMPLE_15 | adeno | 6 | achival | Nivolumab | SD | no | 1.87 | 0.44 | -0.09 | -0.13 | | -0.03 | -0.03 | -0.32 |
| SAMPLE_28 | squamous | 1 | achival | Pembrolizumab | PD | yes | 1.37 | -0.48 | -0.28 | -0.20 | | -0.16 | -0.09 | -0.16 |
| SAMPLE_29 | squamous | 2 | achival | Nivolumab | PD | yes | 0.00 | -0.11 | -0.13 | -0.23 | | -0.16 | -0.07 | -0.24 |
| SAMPLE_30 | squamous | 3 | achival | Nivolumab | SD | yes | 5.57 | 0.69 | -0.15 | -0.05 | | 0.05 | -0.03 | -0.26 |
| SAMPLE_31 | squamous | 1 | achival | Nivolumab | PD | yes | 2.60 | -0.13 | -0.10 | -0.18 | | -0.14 | -0.11 | -0.02 |

PFS, progress free survival; TLS, tumor lymphoid structure; DC, dendritic cell.
