# Supplementary material for: Pan-cancer analysis of genomic properties and clinical outcome associated with tumor tertiary lymphoid structure
Source: Sci Rep. 2020 Dec 9;10:21530. doi: 10.1038/s41598-020-78560-3 (PMC7725838; doi:10.1038/s41598-020-78560-3)
Supplement: Supplementary file 9 — Supplementary Table S3. [file 41598_2020_78560_MOESM9_ESM.docx]

**Supplementary Table S3. Clinical information of Melanoma patients in Riaz. Dataset**

| patient ID | histology | M Stage | biopsy time point | arm | response | live or dead | OS  (weeks) | TLS score | B cells | T cells | CD8 T | Cytotoxic | DC |
| --- | --- | --- | --- | --- | --- | --- | --- | --- | --- | --- | --- | --- | --- |
| Pt10_On_E9047632.6 | melanoma | M1A | on Niv | Nivolumab | SD | dead | 36.60 | 0.07 | -0.01 | -0.03 | 0.22 | 0.04 | 0.03 |
| Pt10_Pre_E9047565.6 | melanoma | M1A | pre Niv | Nivolumab | SD | dead | 36.60 | -0.50 | 0.01 | -0.11 | 0.20 | -0.04 | -0.02 |
| Pt11_On_AD314068.6 | melanoma | NA | on Niv | Nivolumab | PD | dead | 119.60 | 0.49 | 0.02 | 0.11 | 0.22 | 0.16 | 0.16 |
| Pt11_Pre_AD153352.6 | melanoma | NA | pre Niv | Nivolumab | PD | dead | 119.60 | 0.17 | -0.02 | 0.08 | 0.22 | 0.13 | 0.10 |
| Pt13_On_AD314067.6 | melanoma | NA | on Niv | Nivolumab | PD | dead | 40.00 | 0.47 | 0.01 | 0.18 | 0.24 | 0.14 | 0.05 |
| Pt18_On_E9261163.5 | melanoma | M1A | on Niv | Nivolumab | PR | live | 153.30 | 0.27 | 0.31 | 0.37 | 0.30 | 0.29 | 0.07 |
| Pt18_Pre_E9024732.6 | melanoma | M1A | pre Niv | Nivolumab | PR | live | 153.30 | 0.43 | 0.21 | 0.22 | 0.25 | 0.18 | 0.02 |
| Pt24_Pre_AD436687.5 | melanoma | M1C | pre Niv | Nivolumab | PD | live | 21.30 | 0.63 | 0.10 | 0.13 | 0.24 | 0.14 | 0.14 |
| Pt27_On_AD467934.5 | melanoma | M1C | on Niv | Nivolumab | PD | dead | 67.90 | -0.18 | 0.00 | -0.01 | 0.21 | 0.11 | 0.04 |
| Pt27_Pre_AD453873.5 | melanoma | M1C | pre Niv | Nivolumab | PD | dead | 67.90 | -0.36 | 0.02 | -0.03 | 0.22 | 0.09 | -0.07 |
| Pt28_On_AD167375.7 | melanoma | NA | on Niv | Nivolumab | PD | dead | 105.70 | 0.61 | 0.04 | 0.22 | 0.27 | 0.23 | 0.08 |
| Pt28_Pre_AD297619.6 | melanoma | NA | pre Niv | Nivolumab | PD | dead | 105.70 | -0.01 | 0.02 | 0.05 | 0.25 | 0.08 | 0.03 |
| Pt29_Pre_AD497504.5 | melanoma | M1C | pre Niv | Nivolumab | PD | dead | 39.00 | -0.35 | 0.04 | 0.02 | 0.22 | 0.06 | 0.03 |
| Pt3_On_E9047638.6 | melanoma | UNKNOWN | on Niv | Nivolumab | PR | live | 163.40 | 0.30 | 0.01 | 0.02 | 0.23 | 0.08 | 0.11 |
| Pt3_Pre_E9024733.3 | melanoma | UNKNOWN | pre Niv | Nivolumab | PR | live | 163.40 | -0.48 | -0.10 | -0.12 | 0.22 | -0.05 | 0.03 |
| Pt30_On_AD505620.6 | melanoma | M1A | on Niv | Nivolumab | CR | live | 150.40 | 0.82 | 0.05 | 0.34 | 0.29 | 0.26 | 0.12 |
| Pt30_Pre_AD497503.5 | melanoma | M1A | pre Niv | Nivolumab | CR | live | 150.40 | -0.56 | -0.02 | -0.09 | 0.17 | -0.04 | -0.09 |
| Pt31_On_AD467932.6 | melanoma | M1A | on Niv | Nivolumab | PD | live | 137.30 | 0.58 | 0.08 | 0.19 | 0.25 | 0.17 | 0.14 |
| Pt31_Pre_AD453872.5 | melanoma | M1A | pre Niv | Nivolumab | PD | live | 137.30 | -0.03 | 0.05 | 0.10 | 0.24 | 0.10 | 0.13 |
| Pt4_On_E9047643.7 | melanoma | M1B | on Niv | Nivolumab | SD | dead | 90.40 | 0.65 | 0.11 | 0.29 | 0.28 | 0.23 | 0.12 |
| Pt4_Pre_E9021023.6 | melanoma | M1B | pre Niv | Nivolumab | SD | dead | 90.40 | -0.38 | -0.01 | 0.01 | 0.25 | 0.07 | -0.06 |
| Pt44_On_AD167376.6 | melanoma | NA | on Niv | Nivolumab | PR | live | 156.10 | 0.23 | 0.00 | 0.18 | 0.27 | 0.16 | 0.19 |
| Pt44_Pre_AD467790.6 | melanoma | NA | pre Niv | Nivolumab | PR | live | 156.10 | 0.43 | 0.01 | 0.13 | 0.23 | 0.11 | 0.11 |
| Pt5_On_E9047635.6 | melanoma | M1C | on Niv | Nivolumab | PD | dead | 31.70 | 0.01 | -0.05 | 0.03 | 0.21 | 0.10 | -0.02 |
| Pt5_Pre_E9021022.6 | melanoma | M1C | pre Niv | Nivolumab | PD | dead | 31.70 | 0.01 | 0.01 | 0.13 | 0.23 | 0.22 | -0.05 |
| Pt59_Pre_AD823915.5 | melanoma | M1A | pre Niv | Nivolumab | SD | dead | 101.10 | 0.67 | 0.02 | 0.09 | 0.24 | 0.15 | 0.10 |
| Pt62_On_AD635728.5 | melanoma | M1C | on Niv | Nivolumab | PD | dead | 67.40 | -0.51 | -0.07 | -0.09 | 0.20 | -0.01 | 0.09 |
| Pt62_Pre_AD608303.5 | melanoma | M1C | pre Niv | Nivolumab | PD | dead | 67.40 | -0.41 | -0.08 | -0.08 | 0.20 | 0.01 | -0.03 |
| Pt66_Pre_AD667850.6 | melanoma | UNKNOWN | pre Niv | Nivolumab | PD | dead | 77.40 | -0.56 | -0.10 | -0.14 | 0.19 | -0.05 | -0.03 |
| Pt72_Pre_AD793922.5 | melanoma | M1B | pre Niv | Nivolumab | PR | dead | 110.00 | -0.49 | -0.06 | -0.14 | 0.19 | -0.12 | -0.05 |
| Pt77_On_AD733629.8 | melanoma | M1A | on Niv | Nivolumab | SD | dead | 67.30 | 0.41 | 0.27 | 0.36 | 0.31 | 0.25 | 0.22 |
| Pt77_Pre_AD733591.7 | melanoma | M1A | pre Niv | Nivolumab | SD | dead | 67.30 | 0.35 | 0.23 | 0.26 | 0.26 | 0.19 | 0.05 |
| Pt8_On_AD167380.7 | melanoma | M0 | on Niv | Nivolumab | PD | dead | 37.00 | 0.42 | -0.03 | 0.04 | 0.25 | 0.10 | 0.01 |
| Pt8_Pre_AD153354.6 | melanoma | M0 | pre Niv | Nivolumab | PD | dead | 37.00 | -0.01 | 0.02 | 0.04 | 0.24 | 0.08 | 0.05 |
| Pt84_On_AD495199.6 | melanoma | M1C | on Niv | Nivolumab | PD | dead | 21.60 | -0.55 | -0.10 | -0.17 | 0.18 | -0.07 | 0.02 |
| Pt84_Pre_AD486532.5 | melanoma | M1C | pre Niv | Nivolumab | PD | dead | 21.60 | -0.63 | -0.11 | -0.17 | 0.18 | -0.09 | 0.02 |
| Pt87_On_AD495198.6 | melanoma | UNKNOWN | on Niv | Nivolumab | SD | live | 139.10 | 0.78 | 0.16 | 0.32 | 0.29 | 0.26 | 0.18 |
| Pt89_On_AE070987.5 | melanoma | M1A | on Niv | Nivolumab | SD | dead | 120.60 | 0.21 | -0.04 | 0.04 | 0.21 | 0.09 | 0.03 |
| Pt89_Pre_AE070951.5 | melanoma | M1A | pre Niv | Nivolumab | SD | dead | 120.60 | -0.59 | -0.06 | -0.06 | 0.20 | -0.04 | 0.02 |
| Pt9_On_E9047644.7 | melanoma | M1C | on Niv | Nivolumab | PD | dead | 13.10 | -0.73 | -0.08 | -0.10 | 0.23 | -0.10 | 0.06 |
| Pt9_Pre_E9021024.6 | melanoma | M1C | pre Niv | Nivolumab | PD | dead | 13.10 | -0.70 | -0.07 | -0.07 | 0.23 | -0.10 | 0.07 |
| Pt94_On_AE373242.6 | melanoma | M1C | on Niv | Nivolumab | CR | live | 140.10 | 0.50 | -0.02 | 0.04 | 0.21 | 0.12 | 0.05 |
| Pt94_Pre_AD732850.6 | melanoma | M1C | pre Niv | Nivolumab | CR | live | 140.10 | 0.02 | 0.04 | 0.10 | 0.24 | 0.10 | 0.03 |

OS, overall survival; TLS, tumor lymphoid structure; DC, dendritic cell.
