# Supplementary material for: Pan-cancer analysis of genomic properties and clinical outcome associated with tumor tertiary lymphoid structure
Source: Sci Rep. 2020 Dec 9;10:21530. doi: 10.1038/s41598-020-78560-3 (PMC7725838; doi:10.1038/s41598-020-78560-3)
Supplement: Supplementary file 10 — Supplementary Table S4. [file 41598_2020_78560_MOESM10_ESM.docx]

**Supplementary Table S4. Clinical information of SKCM MSKCC cohort**

|  | **OS status** | **OS** | **Sex** | **Age** | **Treatment** | **Durable clinical benefit** | **M stage** | **Tumor site** | **TLS score** | **B cells** | **T cells** | **CD8 T cells** | **Cytotoxic cells** | **DC** |
| --- | --- | --- | --- | --- | --- | --- | --- | --- | --- | --- | --- | --- | --- | --- |
| SD0346 | 1 | 34.75068 | Female | 43 | Ipilimumab  +dacarbazine | LB | M1b | axillary soft tissue metastasis | 0.02 | -0.16 | -0.16 | 0.23 | -0.06 | -0.15 |
| CR6126 | 0 | 22.8 | Female | 66 | Ipilimumab | LB | M1b | elbow metastasis | 0.53 | -0.13 | 0.09 | 0.24 | 0.11 | 0.05 |
| SD6336 | 0 | 83.37534 | Male | 53 | Ipilimumab | LB | M1c | gluteal metastasis | 0.62 | -0.12 | 0.13 | 0.27 | 0.22 | -0.04 |
| LSD0167 | 0 | 32.4 | Male | 33 | Ipilimumab | LB | M1c | small bowel melanoma | 0.68 | -0.04 | 0.05 | 0.25 | 0.17 | 0.00 |
| SD1494 | 0 | 23.70411 | Male | 70 | Ipilimumab | LB | M1c | parietal metastasis | 0.70 | -0.05 | 0.27 | 0.29 | 0.32 | 0.21 |
| CR9699 | 0 | 52.8 | Male | 70 | Ipilimumab | LB | M1c | portal lymph node metastasis | 0.70 | 0.12 | 0.22 | 0.27 | 0.24 | 0.07 |
| CR1509 | 0 | 53.85205 | Female | 54 | Ipilimumab | LB | M1c | gluteal metastasis | 0.71 | -0.05 | 0.27 | 0.28 | 0.27 | 0.20 |
| SD2056 | 0 | 47.24384 | Female | 39 | Ipilimumab | LB | M1b | lung metastasis | 0.73 | 0.04 | 0.20 | 0.30 | 0.27 | 0.05 |
| NR4631 | 1 | 6 | Male | 59 | Ipilimumab | NB | M1c | CNS metastasis | -0.13 | -0.17 | -0.24 | 0.23 | -0.08 | -0.16 |
| SD6494 | 1 | 8.712329 | Female | 63 | Ipilimumab | NB | M1c | small bowel metastasis | -0.09 | -0.17 | -0.17 | 0.20 | -0.17 | -0.13 |
| NR9705 | 1 | 14.4 | Female | 63 | Ipilimumab | NB | M1c | skin/chest wall metastasis | -0.08 | -0.17 | -0.26 | 0.20 | -0.14 | -0.16 |
| NR4810 | 1 | 4.8 | Female | 48 | Ipilimumab | NB | M1c | small bowel metastasis | 0.11 | -0.18 | -0.20 | 0.22 | -0.06 | -0.06 |
| NR9521 | 1 | 32.4 | Male | 74 | Ipilimumab | NB | M1b | L arm melanoma | 0.24 | -0.12 | -0.01 | 0.23 | 0.02 | -0.10 |
| SD5038 | 1 | 13.9726 | Male | 55 | Ipilimumab | NB | M1c | upper back metastasis | 0.36 | -0.14 | -0.10 | 0.23 | 0.05 | 0.04 |
| NR9449 | 1 | 7.2 | Female | 64 | Ipilimumab | NB | M1c | small bowel resection | 0.37 | -0.11 | -0.03 | 0.23 | 0.05 | -0.04 |
| NR3549 | 1 | 6.180822 | Male | 50 | Ipilimumab | NB | M1c | inguinal lymph nodes | 0.39 | -0.07 | 0.01 | 0.23 | 0.03 | 0.01 |
| CR7623 | 0 | 64.8 | Male | 62 | Ipilimumab | NB | M1c | nonresponding adrenal gland metastasis | 0.44 | -0.08 | 0.02 | 0.25 | 0.08 | -0.01 |
| SD5118 | 1 | 32.84384 | Female | 55 | Ipilimumab | NB | M1c | elbow metastasis | 0.57 | 0.13 | 0.18 | 0.26 | 0.15 | 0.04 |
| SD2051 | 1 | 10.1589 | Female | 61 | Ipilimumab  +vemurafenib | NB | M1c | groin lymph node metastases | 0.61 | -0.01 | 0.09 | 0.27 | 0.16 | 0.05 |
| NR5784 | 1 | 5.490411 | Female | 58 | Ipilimumab | NB | M1c | axillary lymph nodes | 0.63 | 0.22 | 0.33 | 0.33 | 0.31 | 0.18 |
| SD7357 | 1 | 24.49315 | Female | 50 | Ipilimumab | NB | M1c | skin and breast metastasis | 0.67 | -0.06 | 0.15 | 0.26 | 0.22 | 0.14 |

OS, overall survival; LB, long-termed benefic, NB, no benefit; TLS, tumor lymphoid structure; DC, dendritic cell.
