# Supplementary material for: Pan-cancer analysis of genomic properties and clinical outcome associated with tumor tertiary lymphoid structure
Source: Sci Rep. 2020 Dec 9;10:21530. doi: 10.1038/s41598-020-78560-3 (PMC7725838; doi:10.1038/s41598-020-78560-3)
Supplement: Supplementary file 11 — Supplementary Table S5. [file 41598_2020_78560_MOESM11_ESM.docx]

**Supplementary Table S5. Clinical information of SKCM DFCI cohort**

|  | OS  status | OS (months) | Sex | Age | Treatment | Durable clinical benefit | M stage | TLS score | B cells | T cells | CD8 T cells | Cytoto-xic cells | DC |
| --- | --- | --- | --- | --- | --- | --- | --- | --- | --- | --- | --- | --- | --- |
| Pat44 | 1 | 9.03333333 | Female | 57 | ipilimumab | PD | M1c | -0.27 | -0.12 | -0.27 | 0.15 | -0.02 | -0.04 |
| Pat45 | 1 | 2.96666667 | Male | 68 | ipilimumab | PD | M1c | -0.19 | -0.10 | -0.28 | 0.13 | -0.09 | -0.10 |
| Pat15 | 1 | 1.66666667 | Male | 32 | ipilimumab | PD | M1c | -0.16 | -0.11 | -0.23 | 0.11 | -0.13 | -0.08 |
| Pat81 | 1 | 20.9333333 | Female | 54 | ipilimumab | PD | M1a | -0.08 | -0.04 | -0.16 | 0.10 | -0.12 | -0.11 |
| Pat41 | 1 | 4.26666667 | Male | 64 | ipilimumab | PD | M1c | -0.06 | -0.05 | -0.11 | 0.15 | -0.13 | 0.02 |
| Pat33 | 1 | 7.03333333 | Male | 65 | ipilimumab | PD | M1c | 0.02 | -0.07 | -0.16 | 0.17 | -0.04 | -0.14 |
| Pat36 | 1 | 1.8 | Female | 52 | ipilimumab | PD | M1c | 0.06 | -0.10 | -0.16 | 0.14 | -0.10 | -0.12 |
| Pat03 | 1 | 3.33333333 | Female | 61 | ipilimumab | PD | M1c | 0.08 | -0.07 | -0.08 | 0.13 | -0.04 | 0.09 |
| Pat50 | 1 | 2.16666667 | Male | 77 | ipilimumab | PD | M1c | 0.09 | -0.06 | -0.06 | 0.11 | -0.01 | -0.02 |
| Pat25 | 1 | 10.8333333 | Male | 69 | ipilimumab | PD | M1c | 0.10 | -0.02 | -0.07 | 0.16 | -0.01 | -0.03 |
| Pat123 | 1 | 28.4333333 | Female | 50 | ipilimumab | SD | M1c | 0.10 | -0.08 | -0.01 | 0.15 | 0.00 | 0.01 |
| Pat86 | 1 | 9.76666667 | Male | 55 | ipilimumab | SD | M1a | 0.11 | -0.05 | -0.08 | 0.17 | 0.00 | -0.04 |
| Pat79 | 1 | 26.7 | Male | 69 | ipilimumab | PR | M1b | 0.14 | -0.03 | 0.00 | 0.13 | 0.01 | -0.04 |
| Pat119 | 0 | 26.9333333 | Male | 61 | ipilimumab | PD | M0 | 0.18 | -0.01 | 0.02 | 0.14 | 0.01 | 0.06 |
| Pat90 | 0 | 33.5 | Male | 59 | ipilimumab | PR | M1c | 0.18 | -0.03 | 0.04 | 0.17 | 0.06 | -0.07 |
| Pat46 | 1 | 5.33333333 | Female | 36 | ipilimumab | PD | M1b | 0.20 | -0.04 | -0.09 | 0.15 | -0.02 | -0.12 |
| Pat40 | 1 | 1.13333333 | Male | 74 | ipilimumab | PD | M1c | 0.20 | -0.07 | -0.08 | 0.13 | -0.06 | 0.03 |
| Pat37 | 1 | 2.33333333 | Female | 47 | ipilimumab | PD | M1c | 0.21 | -0.06 | 0.03 | 0.12 | -0.02 | 0.02 |
| Pat47 | 0 | 36.8666667 | Male | 78 | ipilimumab | CR | M1c | 0.22 | -0.06 | -0.15 | 0.13 | -0.04 | -0.02 |
| Pat27 | 0 | 45.9666667 | Male | 61 | ipilimumab | PD | M1c | 0.23 | -0.05 | -0.03 | 0.14 | -0.01 | -0.06 |
| Pat16 | 1 | 27.0333333 | Female | 68 | ipilimumab | PD | M1b | 0.24 | 0.00 | 0.12 | 0.14 | 0.05 | 0.00 |
| Pat14 | 1 | 5.43333333 | Female | 48 | ipilimumab | PD | M1c | 0.24 | -0.03 | 0.04 | 0.19 | 0.07 | 0.04 |
| Pat04 | 0 | 32.9 | Male | 71 | ipilimumab | PR | M1b | 0.26 | -0.05 | 0.00 | 0.13 | 0.04 | -0.02 |
| Pat98 | 1 | 4.66666667 | Female | 57 | ipilimumab | PD | M1c | 0.30 | -0.02 | 0.00 | 0.13 | -0.02 | -0.04 |
| Pat29 | 0 | 44.2 | Male | 82 | ipilimumab | X | M1c | 0.30 | 0.15 | 0.17 | 0.16 | 0.11 | 0.05 |
| Pat02 | 0 | 54.4 | Female | 42 | ipilimumab | SD | M1c | 0.31 | -0.05 | 0.16 | 0.21 | 0.14 | 0.14 |
| Pat83 | 0 | 34.2333333 | Male | 22 | ipilimumab | PD | M1c | 0.32 | -0.01 | 0.18 | 0.19 | 0.18 | -0.05 |
| Pat39 | 0 | 49.5666667 | Male | 67 | ipilimumab | CR | M1b | 0.33 | 0.11 | 0.17 | 0.18 | 0.13 | 0.22 |
| Pat80 | 1 | 24.1333333 | Male | 48 | ipilimumab | SD | M1c | 0.34 | 0.00 | 0.08 | 0.15 | 0.06 | -0.06 |
| Pat126 | 0 | 21.3666667 | Male | 77 | ipilimumab | PR | M1b | 0.37 | -0.05 | 0.04 | 0.13 | 0.01 | 0.03 |
| Pat118 | 1 | 10.4333333 | Female | 43 | ipilimumab | PD | M1c | 0.41 | -0.02 | 0.16 | 0.16 | 0.10 | 0.19 |
| Pat08 | 1 | 4.66666667 | Male | 73 | ipilimumab | PD | M1c | 0.42 | -0.03 | 0.08 | 0.14 | 0.05 | 0.07 |
| Pat19 | 1 | 5.83333333 | Male | 59 | ipilimumab | PD | M1c | 0.43 | 0.02 | 0.13 | 0.16 | 0.10 | 0.14 |
| Pat28 | 1 | 39.4666667 | Male | 77 | ipilimumab | PD | M1c | 0.45 | 0.01 | 0.13 | 0.20 | 0.13 | -0.02 |
| Pat88 | 0 | 32.9666667 | Female | 60 | ipilimumab | SD | M1c | 0.46 | 0.06 | 0.23 | 0.22 | 0.22 | -0.04 |
| Pat43 | 1 | 1.23333333 | Female | 75 | ipilimumab | PD | M1b | 0.46 | 0.00 | 0.24 | 0.21 | 0.16 | 0.13 |
| Pat06 | 1 | 5.36666667 | Male | 33 | ipilimumab | PD | M1c | 0.52 | 0.11 | 0.21 | 0.18 | 0.14 | 0.22 |
| Pat38 | 0 | 51.3 | Male | 45 | ipilimumab | PR | M1c | 0.55 | 0.00 | 0.21 | 0.18 | 0.18 | 0.15 |
| Pat49 | 0 | 34.4666667 | Male | 36 | ipilimumab | SD | M1c | 0.55 | 0.07 | 0.27 | 0.20 | 0.24 | 0.00 |
| Pat85 | 1 | 15.2666667 | Male | 83 | ipilimumab | PD | M1c | 0.58 | 0.04 | 0.32 | 0.22 | 0.24 | -0.04 |

OS, overall survival; TLS, tumor lymphoid structure; DC, dendritic cell.
